# Comparative effectiveness of acupuncture-related therapies for frozen shoulder: a systematic review and network meta-analysis

**DOI:** 10.3389/fmed.2025.1673193

**Published:** 2025-11-26

**Authors:** Rongyao Ji, Wenrui Huang, Mingjie Weng, Min Zhang

**Affiliations:** 1 Shanxi University of Chinese Medicine, Taiyuan, Shanxi, China; 2 Shenzhen Traditional Chinese Medicine Hospital, Shenzhen, China; 3 Department of Sports and Joint Surgery, Second Hospital of Shanxi Medical University, Taiyuan, Shanxi, China

**Keywords:** frozen shoulder, acupuncture, physiotherapy, effectiveness, network meta-analysis

## Abstract

**Background:**

Frozen shoulder is a common condition that limits shoulder mobility and causes pain, significantly affecting daily function. Acupuncture and related therapies are increasingly used as treatment options and may offer potential benefits, but their overall effectiveness remains uncertain. This study aims to systematically evaluate the clinical efficacy of acupuncture and related therapies for frozen shoulder through a network meta-analysis.

**Methods:**

We searched Chinese and international databases, including PubMed, Cochrane Library, EMbase, Web of Science, China National Knowledge Infrastructure, Wanfang Database, VIP Database, and China Biomedical Literature Database, for randomized controlled trials (RCTs) published from database inception to September 2025 on acupuncture and related therapies for Frozen Shoulder. Eligible studies were screened, and two independent reviewers assessed the quality and risk of bias using the ROB 2 tool from the Cochrane Handbook. Data were analyzed using Stata 18.0 software for network meta-analysis. The relative efficacy of each intervention was ranked using the surface under the cumulative ranking curve (SUCRA).

**Results:**

A total of 84 RCTs involving 7,125 patients and 17 interventions were included. For the overall effectiveness rate, small needle knife therapy showed the greatest improvement over both physical therapy [relative risk (RR) = 1.39, 95% confidence interval (CI) 1.21–1.60] and Western medicine (RR = 1.39, 95% CI 1.22–1.58). In terms of the apparent healing rate, joint mobilization combined with warm acupuncture-moxibustion achieved the most pronounced benefit compared with physical therapy (RR = 1.83, 95% CI 1.19–2.83) and Western medicine (RR = 2.19, 95% CI 1.36–3.54). For shoulder function, measured by the Constant–Murley Shoulder (CMS) score, floating needle therapy yielded the largest functional gain relative to physical therapy [standardized mean difference (SMD) = 3.12, 95% CI 1.91–4.33] and Western medicine (SMD = 4.87, 95% CI 3.47–6.26). Concerning pain intensity, assessed by the Visual Analogue Scale (VAS), Western medicine provided slightly greater pain reduction than acupuncture-based interventions, though the differences were not statistically significant. Adverse events were infrequent and generally mild, occurring less often in acupuncture-related therapies than in control groups. Across outcomes, the certainty of evidence ranged from low to moderate.

**Conclusion:**

Small needle knife therapy, joint mobilization plus warm acupuncture-moxibustion, and massage combined with acupuncture appear most effective for improving outcomes in frozen shoulder. Floating needle and moxibustion show advantages in restoring shoulder function. Acupuncture-based therapies are generally safe, but the overall evidence is of moderate-to-low certainty, warranting further high-quality, multicenter trials.

**Systematic review registration:**

CRD42024610867.

## Introduction

Adhesive capsulitis, commonly referred to as frozen shoulder, is a disabling musculoskeletal disorder characterized by pain, progressive stiffness, and restricted range of motion across multiple planes (1). The condition may be idiopathic or secondary to systemic or local factors such as diabetes, thyroid disease, or prolonged immobilization (1). Pathophysiologically, frozen shoulder is marked by synovial inflammation followed by capsular fibrosis and contracture, leading to a protracted clinical course that can last months to years and often results in substantial functional impairment (2, 3). The burden of frozen shoulder is considerable. It affects 3–5% of the general population, most frequently between the ages of 40 and 65, with a higher incidence in women (1). Individuals with diabetes are particularly vulnerable, with prevalence rates of 10–20% (1). Beyond its clinical manifestations, frozen shoulder imposes a significant socioeconomic cost through prolonged disability, extended sick leave, and high medical expenditures (4). For instance, patients with post-traumatic adhesive capsulitis may require work leave up to seven times longer than those with uncomplicated shoulder injuries (4). Given this protracted course and the high economic burden, reliance on “watchful waiting” is often clinically untenable (5).

Current treatment approaches focus on pain control and restoration of mobility, yet no consensus exists regarding the optimal evidence-based strategy (2). Conservative measures such as physical therapy are widely recommended but supported by heterogeneous and often low-quality evidence (6, 7). Corticosteroid injections can provide short-term pain relief but carry local and systemic risks, while surgical interventions such as manipulation under anesthesia or arthroscopic capsular release are typically reserved for refractory cases and are associated with potential complications (5, 8). Thus, there is a pressing need for safe, minimally invasive alternatives that can accelerate recovery without imposing additional risk.

Acupuncture-related therapies (ARTs), including manual acupuncture, electroacupuncture, moxibustion, and other adjunctive techniques, are increasingly recognized as non-pharmacological interventions for musculoskeletal pain. Evidence suggests that ARTs exert their effects through modulation of inflammatory pathways, neuroimmune regulation, and improvement of local circulation (9, 10). Despite encouraging findings, the diversity of acupuncture modalities raises uncertainty about their comparative effectiveness.

Several systematic reviews have examined this question. Xu et al. reported that acupuncture combined with physical therapy improved pain and function, but their review focused on combined interventions rather than individual modalities (11). Wang et al. evaluated warm needle therapy and suggested potential benefit, though the review protocol did not provide comprehensive comparative evidence (12). Ben-Arie et al. confirmed that acupuncture was more effective than control treatments, yet substantial heterogeneity remained and no clear ranking of interventions was established (3). Collectively, these reviews support the potential value of ARTs but leave unresolved which specific modalities are most effective.

Conventional pairwise meta-analyses therefore cannot fully meet clinical needs, as they are limited to direct comparisons and lack the capacity to rank competing therapies. Network meta-analysis provides a robust approach by integrating direct and indirect evidence, allowing simultaneous comparison of multiple interventions within a single framework. The present study aims to synthesize available randomized controlled trials (RCTs) to evaluate and rank the relative efficacy and safety of ARTs for frozen shoulder, thereby providing evidence-based guidance for clinical decision-making and policy.

## Methods

This systematic review and network meta-analysis was conducted in accordance with the PRISMA 2020 guidelines (13) and the PRISMA extension statement for network meta-analyses (PRISMA-NMA) (14) to ensure methodological transparency and completeness (Appendix 1). The protocol was prospectively registered in PROSPERO (CRD42024610867).

### Search strategy

We systematically searched both English and Chinese databases, including PubMed, Cochrane Library, Embase, Web of Science, China National Knowledge Infrastructure (CNKI), Wanfang, VIP, and SinoMed, from their inception to September 2025. The search strategies combined Medical Subject Headings (MeSH) and free-text terms related to frozen shoulder and acupuncture-related therapies (e.g., “acupuncture,” “electroacupuncture,” “moxibustion,” “fire needle,” “warm acupuncture-moxibustion,” “small needle knife,” “floating needle,” and “bianshi therapy”). Boolean operators (AND, OR) and truncation were used as appropriate to ensure sensitivity and specificity of the searches. No restrictions were applied regarding publication year. Studies published in English and Chinese were included. Reference lists of relevant systematic reviews and included trials were manually screened to identify additional eligible studies. Gray literature, including conference proceedings and dissertations, was also considered where accessible. Detailed search strategies for each database, including both English and Chinese queries, are provided in Appendix 2.

### Eligibility criteria

We included RCTs evaluating acupuncture or related therapies for frozen shoulder. Patients were eligible if they had a confirmed diagnosis of frozen shoulder, as defined by the diagnostic criteria of the Chinese Medical Association (CMA) (15), regardless of age, sex, or race. A summary of the CMA diagnostic criteria is presented in Appendix Table 3.2. Eligible interventions for the experimental groups included manual acupuncture, electroacupuncture, moxibustion, fire needle, small needle knife, floating needle, and Bianshi therapy, as well as combined approaches (e.g., acupuncture plus massage, joint mobilization plus warm acupuncture-moxibustion). Control groups received Western medicine or physiotherapy. In multi-arm trials, at least two study arms had to meet these criteria, and comparisons between different acupuncture-related therapies were also considered eligible for inclusion.

The primary outcomes were total clinical effectiveness rate, Visual Analogue Scale (VAS) for pain, and cure–marked improvement rate. Secondary outcomes included the Constant–Murley Shoulder (CMS) score and adverse events or withdrawal rates. The primary outcomes were total clinical effectiveness rate, VAS for pain, and cure–marked improvement rate. Secondary outcomes included the CMS score, and Adverse reactions and shedding. Specifically, clinical cure was defined as disappearance or near disappearance of shoulder pain, stiffness, and movement limitation, with an improvement rate of ≥95%; markedly effective as 70–94% improvement; effective as 30–69% improvement; and ineffective as <30% improvement. The total effectiveness rate was calculated as the proportion of patients classified as cured, markedly effective, or effective out of the total number of cases, while the cure–marked improvement rate referred to the proportion of patients who were cured or markedly effective. These standardized definitions were consistently applied across all included studies to enhance comparability and interpretability of outcomes.

Studies were excluded if they contained duplicate data, provided insufficient methodological or outcome details, were not published in Chinese or English, involved animal experiments, or were available only as conference abstracts.

### Data extraction

Two independent reviewers screened titles, abstracts, and full texts of potentially eligible studies. Data from included trials were extracted into a standardized Excel spreadsheet, including study title, publication year, participant characteristics (age, sample size, disease duration), intervention details, treatment regimens, and outcome measures. After extraction, the two reviewers cross-checked the data. Any discrepancies were resolved through discussion, with arbitration by a third reviewer when necessary.

### Risk of bias and certainty of evidence

Risk of bias was independently assessed by two reviewers using the Cochrane Risk of Bias 2 (RoB 2) tool (16), which evaluates random sequence generation, deviations from intended interventions, missing outcome data, outcome measurement, selective reporting, and overall risk of bias. Disagreements were resolved through discussion, with a third reviewer consulted when consensus could not be reached. The certainty of evidence was further appraised using the CINeMA (Confidence in Network Meta-Analysis) framework. This approach considers six domains: within-study bias, reporting bias, indirectness, imprecision, heterogeneity, and inconsistency. Intransitivity was explored by comparing potential effect modifiers—such as baseline age, disease duration, and intervention type—across studies contributing direct and indirect evidence to each comparison (17, 18).

### Statistical analysis

We conducted the network meta-analysis within a frequentist framework using Stata 18.0 and the network and mvmeta packages (19, 20). A random-effects model was applied to account for between-study heterogeneity. For continuous outcomes, mean difference (MD) with 95% confidence intervals (CIs) was calculated; for dichotomous outcomes, relative risk (RR) with 95% CIs was used. Results were considered statistically nonsignificant when the CI contained the null value (0 for MD, 1 for RR) (21). Network plots were constructed to illustrate the evidence base, with node size indicating the number of participants and edge thickness representing the number of direct comparisons. Forest plots summarized pooled estimates. Between-study heterogeneity was quantified using τ^2^, classified as low (<0.04), low–moderate (0.04–0.16), moderate–high (0.16–0.36), or high (>0.36) according to established thresholds (22–24). A common τ^2^ was assumed across all contrasts, with a correlation of 0.5 specified in the between-study covariance matrix. Consistency between direct and indirect evidence was examined using the node-splitting method, which separates sources of evidence within each closed loop of the network (25). Significant discrepancies were considered indicative of inconsistency and informed the overall interpretation of robustness. Potential publication bias and small-study effects were assessed using comparison-adjusted funnel plots; asymmetry was interpreted as possible systematic bias. Intervention rankings were derived from the surface under the cumulative ranking curve (SUCRA), which ranges from 0 to 100%, with higher values indicating a greater probability of being among the most effective treatments (26).

## Results

### Study selection

**Figure 1:**
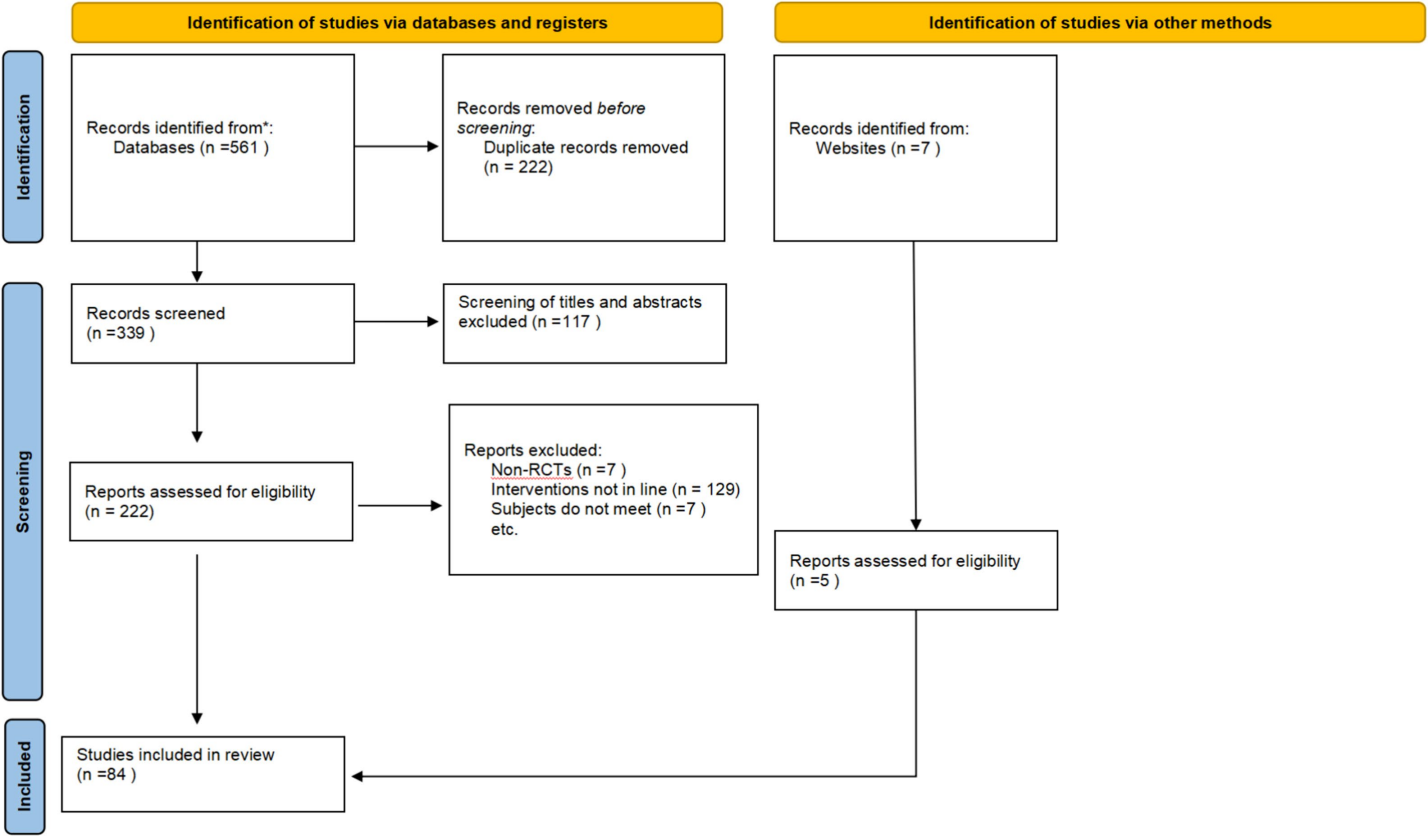
Literature search flowchart of included studies.

The initial search identified 561 records. After removal of duplicates, 339 unique articles remained. Screening of titles and abstracts excluded 117studies, leaving 222 for full-text review. Of these, 143 were excluded for reasons such as mismatched diagnostic criteria, ineligible interventions or outcomes, insufficient data, or duplicate publication. Consequently, 79 RCTs were included in the analysis. An updated search was subsequently performed to ensure completeness, yielding 5 additional eligible studies. In total, 84 RCTs were included in the final network meta-analysis. The study selection process is illustrated in Figure 1.

### Characteristics of included studies

A total of 84 RCTs (27–95) involving 7,125 participants were included, comprising 3,677 patients in the intervention groups and 3,448 in the control groups. Sample sizes across studies ranged from fewer than 30 to more than 200 participants per trial. The mean age of participants was generally between 40 and 65 years, with several studies reporting elderly cohorts above 70 years. Most participants had a disease duration of within 1 year, while a small number of patients had a disease history of more than 5 years. The interventions covered 17 types of acupuncture-related therapies, including manual acupuncture, electroacupuncture, moxibustion, warm acupuncture–moxibustion, fire needle, floating needle, small needle knife, Bianshi therapy, acupuncture combined with cupping, massage combined with acupuncture, and joint mobilization alone or in combination with acupuncture-related techniques. Control groups primarily received Western medicine or physical therapy, though several studies also compared different acupuncture modalities directly. Treatment duration ranged from 1 to 90 days, with most trials administering interventions over 14–28 days (Table 1).

**Table 1:** Basic research characteristics of the included literature.

Author/Year	Number (T)	Number(C)	Age(T)/years	Age(C)/years	Duration of disease(T)/month	Duration of disease(C)/month	I-Treatment	I-Control	Course of treatment/Days	Outcome
Yang et al. (29)	125	125	60.50 ± 7.75	64.00 ± 8.00			E	D	21d	①②
Xingang (30)	51	51	73.2 ± 5.8	71.6 ± 5.8			C	P	41d	①
Tao et al. (31)	30	30					B	N	14d	①
Zhiwu and Hongping (32)	30	30					E	C	7d	①②
Huizhen et al. (33)	64	43					D	C	22d	①
Libin et al. (34)	40	40	48.1 ± 2.2	47.8 ± 2.3			M	C	28d	①②
Li (35)	38	38					D	B	20d	①②④
Yingchun et al. (36)	43	43	49.6 ± 4.3	48.9 ± 4.1	3.3 ± 1.4	3.2 ± 1.5	D	A	20d	①②④
Yong et al. (37)	40	30					F	A	14d	①④
Yanxi and Peng (38)	100	90					E	A	10d	①
Liqun and Mingbo (39)	80	52	49 ± 6.1	51 ± 4.8			M	N	14d	②
Chunhai (40)	41	41	55.26 ± 9.19	56.13 ± 9.21	1.36 ± 0.57	1.55 ± 0.47	D	A	7d	①
Jiagui (41)	40	40	52.23 ± 8.75	54.18 ± 7.46	24.56 ± 9.23	17.62 ± 10.57	E	O	30d	①
Zhengen et al. (42)	90	90	54 ± 16	55 ± 17	108.3 ± 18.2	110.5 ± 15.9	J	A	30d	①②
Chenglin et al. (43)	90	60	57.37 ± 7.16	57.55 ± 9.40			C	N	14d	①②⑤
Chenglin et al. (43)	90	60	58.34 ± 7.78	57.55 ± 9.40			P	N	14d	①②⑤
Jingyu (44)	40	40					A	P	10d	①④
Changqing et al. (45)	120	120					H	B	15d	②③
Li et al. (46)	62	61	44.12 ± 3.42	41.37 ± 4.10	3.18 ± 1.01	4.00 ± 0.98	E	B	21d	①②③
Yucai (47)	25	20	52.68 ± 6.11	53.79 ± 5.27			I	P	7d	①②④⑤
Rongjuan et al. (48)	45	45	58.64 ± 5.21	57.96 ± 4.78	1.25 ± 0.31	1.33 ± 0.47	E	A	20d	①④
Ping et al. (49)	46	46					B	N	6d	③
Shuirong (50)	40	40	53.57 ± 11.29	55.28 ± 10.61	3.15 ± 0.48	3.25 ± 0.56	D	C	26d	①②④
Lianxin et al. (51)	30	30	53.2 ± 3.9	53.2 ± 3.9	3.1 ± 1.3	3.1 ± 1.3	E	O	7d	①④
Zhao (52)	70	70	57.33 ± 8.07	55.27 ± 9.41	98.6 ± 49.7	100.8 ± 58.4	A	C	42d	①
Zhao (52)	75	70	56.25 ± 6.72	55.27 ± 9.41	94.8 ± 46.5	100.8 ± 58.4	M	C	42d	①
Jinguo (53)	43	43	48.96 ± 4.84	48.35 ± 4.71	1.64 ± 0.47	1.57 ± 0.41	D	O	10d	①④
Decong et al. (54)	41	41	4.98 ± 11.15	55.86 ± 10.73	9.03 ± 4.05	8.97 ± 3.52	L	D	7d	①②④
Peizheng (55)	60	60	48.7 ± 2.4	48.8 ± 2.6	4.9 ± 2.0	4.8 ± 2.1	C	B	56d	①②③④
Junhe et al. (56)	31	31	49.65 ± 3.56	48.82 ± 3.16	2.38 ± 0.5	2.78 ± 0.65	G	E	28d	②
Xinwei et al. (57)	40	40	42.1 ± 9.2	44.4 ± 8.6	12.8 ± 10.9	13.2 ± 9.0	I	A	24d	①②④
Yan and Huagong (58)	112	56					D	P	22d	①④
Linyan et al. (59)	40	40	57.27 ± 6.82	55.40 ± 6.98	52.93 ± 17.36	52.05 ± 17.55	B	A	14d	①②⑤
Ziling et al. (61)	63	63	49.2 ± 6.3	49.3 ± 6.1	6.8 ± 4.2	6.5 ± 4.4	L	A	20d	①④
Jianwei (62)	80	80	55.62 ± 8.32	55.62 ± 8.32	12 ± 7. 5	12 ± 7. 5	C	A	28d	①④
Ming ad Litao (63)	50	50	51. 36 ± 1.43	52. 53 ± 1.31	1. 05 ± 0. 23	1. 04 ± 0. 35	L	G	23d	①②
Wei (68)	40	40	50.2 ± 6.2	50.2 ± 6.2	17.4 ± 5.2	17.4 ± 5.2	L	G	34d	①④
Yuanyuan et al. (65)	30	30	48.93 ± 14.20	50.68 ± 14.87	5.36 ± 0.32	5.48 ± 0.38	O	A	22d	①
Weiping et al. (66)	45	45					D	Q	15d	①④
LiHui et ql. (67)	45	45	50.62 ± 3.37	50.49 ± 3.21	11.36 ± 3.23	11.13 ± 3.52	D	P	42d	①④
Wei (68)	36	36	56.1 ± 2.5	57.2 ± 2.3			F	A		①④
Shangxi (69)	35	35	53.44 ± 3.13	54.33 ± 3.41	1.29 ± 0.34	1.36 ± 0.31	D	N	28d	①②④
Ye and Long (70)	41	41					K	N	15d	①④⑤
Hui et al. (72)	57	56	54.86 ± 1.06	52.14 ± 0.92	3.11 ± 0.30	2.97 ± 0.33	B	A	10d	①②④
Hui et al. (72)	61	56	54.18 ± 1.00	52.14 ± 0.92	3.00 ± 0.47	2.97 ± 0.33	D	A	10d	①②④
Biaomin and Jinxiong (73)	68	42					G	A	10d	①
Chenyao et al. (74)	50	47	54.08 ± 7.97	55.13 ± 9.14	39.72 ± 44.14	40.12 ± 36.77	B	N	7d	①②④
Fengchuan (75)	50	50					E	O	30d	①④
Hongwei and Maifang (76)	50	50					F	A	7d	①
Hongguo (77)	31	31	48.41 ± 4.21	44.51 ± 7.67	13.51 ± 5.26	11.54 ± 6.25	J	A	14d	②
Hui e tal. (72)	30	30	59.07 ± 10.94	58.96 ± 11.47	1.64 ± 0.33	1.67 ± 0.27	J	A	14d	①②
Xi et al. (79)	32	32	54.81 ± 1.76	58.34 ± 1.73	12.47 ± 1.36	14.19 ± 7.29	I	B	21d	①②④⑤
Bin (80)	44	44	54.52 ± 3.43	54.43 ± 3.36			A	Q	14d	①④
Chengju et al. (81)	54	54					M	C	10d	①
Guowei (82)	30	30					G	P	20d	①②④
Xianzhao et al. (83)	50	50	53.71 ± 3.95	52.44 ± 3.75	3.54 ± 2.45	3.24 ± 1.51	F	A	19d	①②④
Xiangwei (84)	30	30	55.37 ± 5.29	54.60 ± 5.24	5.97 ± 2.44	6.20 ± 2.20	F	A	28d	①②
Kaisheng et al. (85)	43	43	52∙47 ± 10∙85	51∙66 ± 12.58	82.62 ± 40.3	78.88 ± 39.7	K	B	12d	①②④
Hongwei (86)	50	50	55.5 ± 17.6	55.8 ± 17.3			D	N	21d	①④
Xuejun (87)	68	68	54.5 ± 5.6	54.3 ± 5.3	1.2 ± 0.8	1.1 ± 0.7	A	C	15d	①④⑤
Guanghao et al. (88)	46	46					B	N	15d	①④
Liang (87)	33	32					A	N	28d	①③④
Ruilian (90)	72	72	49. 5 ± 5.86	49. 7 ± 5. 98	4. 67 ± 2. 95	4. 54 ± 2.78	E	A	10d	①②④
Minming (91)	42	42	66.3 ± 7.2	67.1 ± 6.5	1.6 ± 0.4	1.7 ± 0.3	L	G	20d	①②④
Tian et al. (92)	31	34					F	A	28d	①②③④
Xiaoping (93)	41	37	46.27 ± 9.15	44.85 ± 8.21	21.86 ± 15.67	23.52 ± 12.69	D	B	15d	①②④
Zhou et al. (94)	31	35	51 ± 7.41	50 ± 9.63	2 ± 3.85	3.5 ± 3.7	F	A	28d	①②③④
Wang et al. (95)	36	36	51.47 ± 3.89	51.47 ± 3.89	8.33 ± 4.21	9.27 ± 4.77	L	G	21d	①④
Wang et al. (96)	30	30	53 ± 8	53 ± 9	6.90 ± 1.02	6.74 ± 1.04	A	N	10d	①②④
Wang et al. (96)	30	30	53 ± 8	53 ± 9	6.83 ± 1.06	6.74 ± 1.04	K	N	10d	①②④
Lu et al. (97)	30	30	55.27 ± 11.69	54.8 ± 9.03	4.96 ± 2.75	4.47 ± 2.92	I	A	1d	①②④
Chen et al. (98)	40	40	58.4 ± 6.75	59 ± 6.75			B	C	24d	①②③④
Chen et al. (98)	40	40	57.2 ± 5.75	59 ± 6.75			P	C	24d	①②③④

A, Acupuncture; B, Electroacupuncture; C, Massage; D, Warm Acupuncture-moxibustion; E, Small Needle Knife; F, Acupuncture and Cupping Therapy; G, Joint Mobilization; H, Bianshi Therapy; I, Floating Needle; J, Fire Meedling; K, Moxibustion; L, Joint Mobilization + Warm Acupuncture-Moxibustion; M, Massage+Acupuncture; N, Western Medicine; O, Acupoint Injection; P, Physical Therapy; Q, Functional Exercise; ① Overall effectiveness rate; ② VAS Score; ③ Adverse reactions and shedding.; ④ Apparent Healing Rate; ⑤ CMS score.

### Risk of bias, certainty of evidence, and consistency

**Figure 2:**
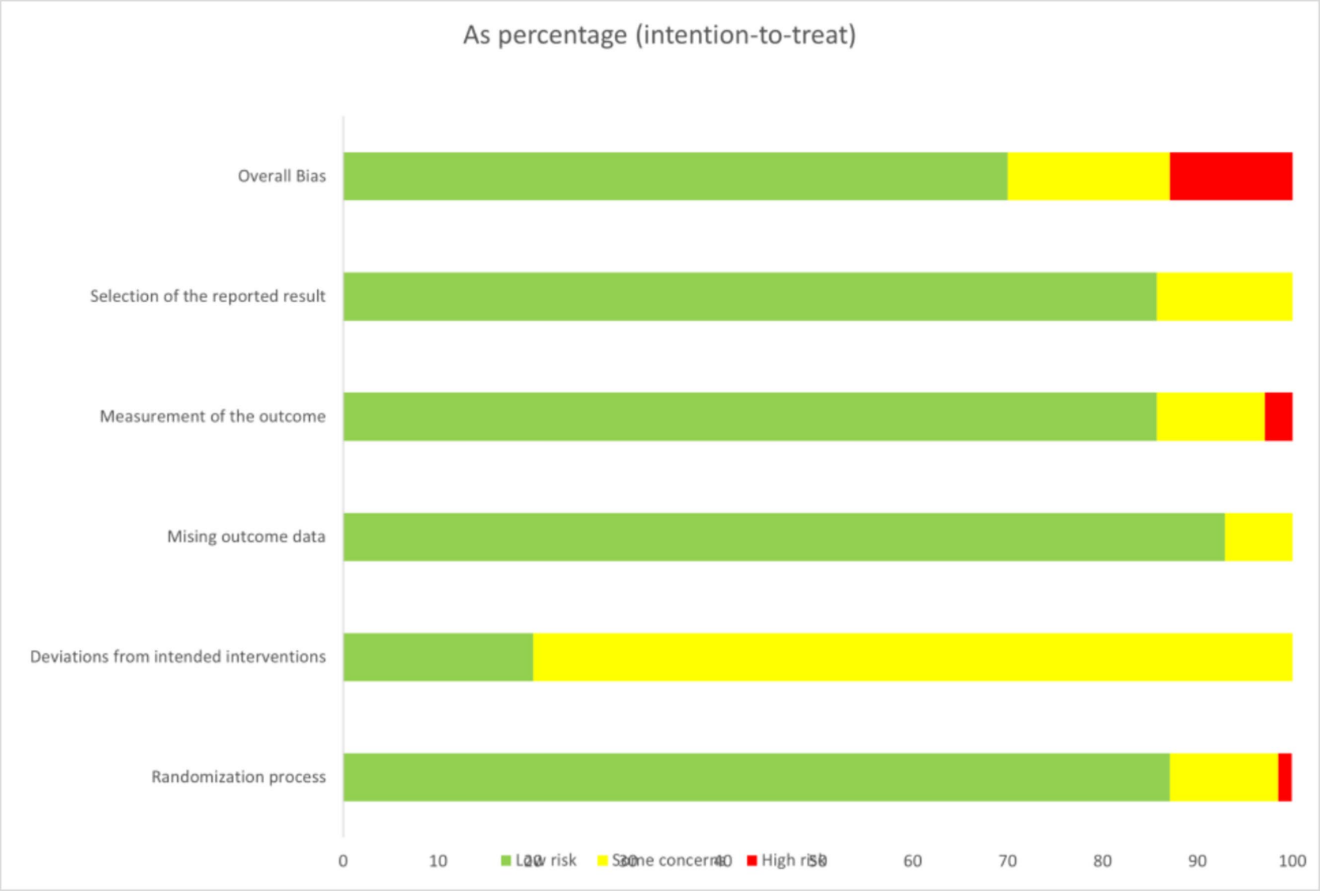
Percentage of items with risk of bias in the included studies.

Risk of bias was assessed using the RoB 2 tool. Most trials were judged as having some concerns, mainly due to insufficient reporting of blinding and deviations from intended interventions. Randomization procedures and outcome measurement were generally adequate and rated as low risk, while missing data and selective reporting were rarely problematic. A minority of trials were rated as high risk owing to poor methodological descriptions. Overall, the body of evidence was of moderate quality, as shown in Figure 2 and detailed in Appendix 4. Consistency assessments revealed no significant global inconsistency across the networks (Appendix Table S5.1). Node-splitting analyses further confirmed that all comparisons had *p* values >0.05, indicating no local inconsistency (Appendix Table S5.2). Between-study heterogeneity was generally low to moderate (τ^2^ < 0.16), with no evidence of high heterogeneity across comparisons. Certainty of evidence, assessed using the CINeMA framework, was rated as low to moderate across most outcomes, reflecting concerns mainly related to reporting quality and indirectness (Appendix 6). Funnel plots did not reveal notable asymmetry, suggesting no evidence of publication bias (Appendix 7).

### Overall effectiveness rate

**Figure 3:**
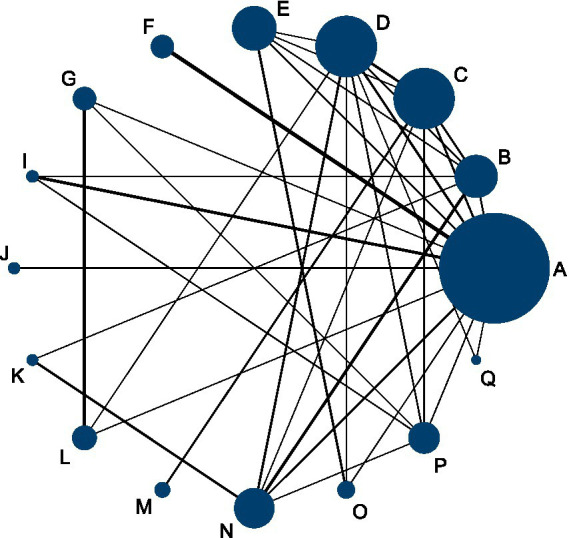
Network diagram comparing the overall effectiveness rate of acupuncture and related therapies for frozen shoulder.

This NMA evaluated improvements in overall effectiveness rate across 68 RCTs involving 6,536 participants. As shown in Figure 3, the network included both direct and indirect comparisons between acupuncture-related interventions and control treatments—physical therapy (P) and Western medicine (N). The thickness of each connecting line in the network plot indicates the frequency of direct comparisons, with acupuncture combined with cupping (F) and Western medicine (N) appearing most frequently.

**Figure 4:**
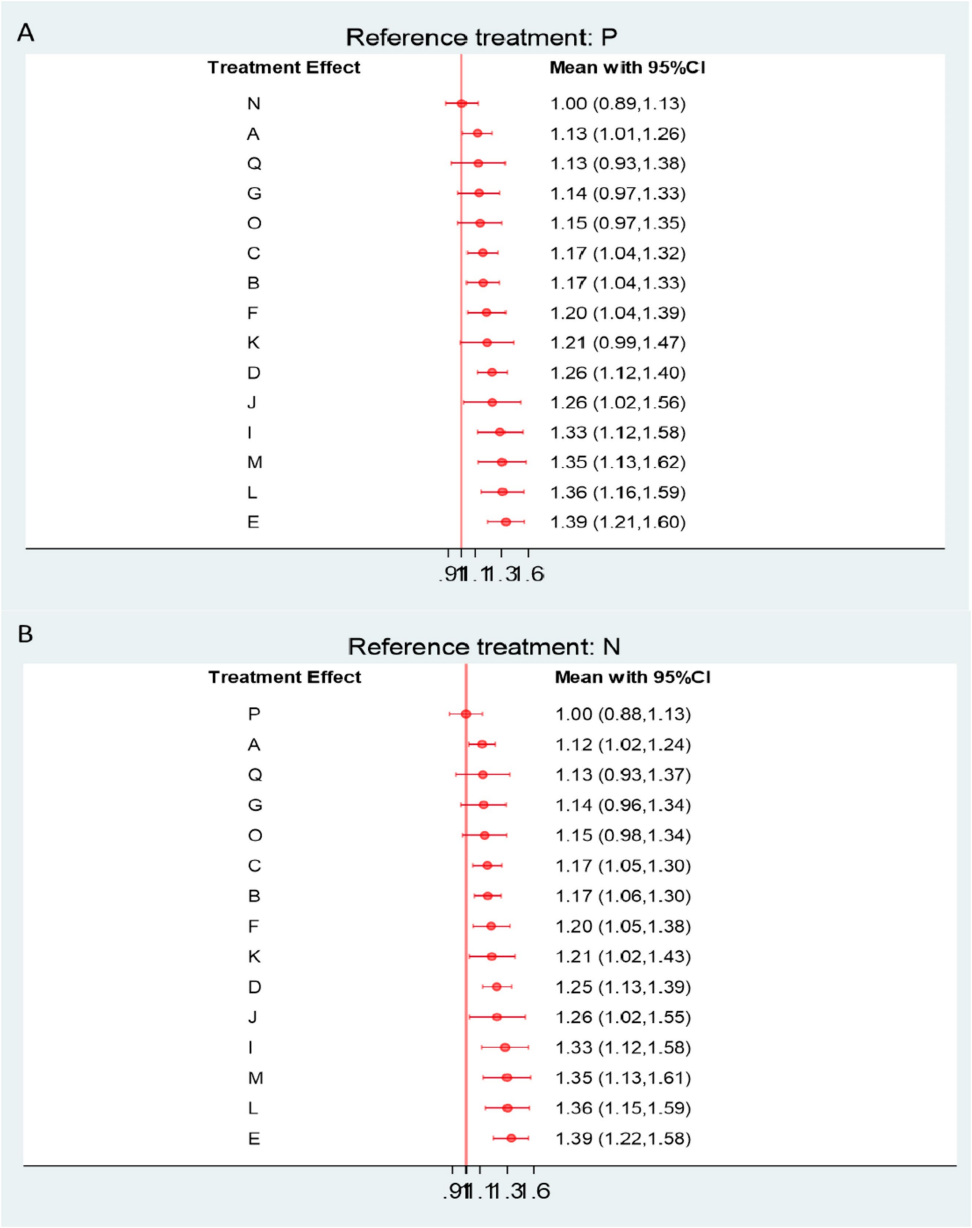
**(A)** Forest plot comparing the overall effectiveness rate of acupuncture and related therapies versus physical therapy. **(B)** Forest plot comparing the overall effectiveness rate of acupuncture and related therapies versus Western medicine. A, Acupuncture; B, Electroacupuncture; C, Massage; D, Warm Acupuncture-moxibustion; E, Small Needle Knife; F, Acupuncture and Cupping Therapy; G, Joint Mobilization; H, Bianshi Therapy; I, Floating Needle; J, Fire Meedling; K, Moxibustion; L, Joint Mobilization + Warm Acupuncture-Moxibustion; M, Massage+Acupuncture; N, Western Medicine; O, Acupoint Injection; P, Physical Therapy; Q, Functional Exercise.

Forest plots demonstrated that, among patients with frozen shoulder, all acupuncture-related interventions achieved higher overall effectiveness than either physical therapy or Western medicine. Specifically, nine interventions showed significant improvement compared with physical therapy (Figure 4A), and 10 interventions were superior to Western medicine (Figure 4B). Among these, small needle knife therapy (E) produced the greatest improvement in total effectiveness (vs. physical therapy: RR = 1.39, 95% CI 1.21–1.60, SUCRA = 93.6%, low certainty; vs. Western medicine: RR = 1.39, 95% CI 1.22–1.58, SUCRA = 93.6%, low certainty). Comparable benefits were observed for joint mobilization plus warm acupuncture-moxibustion (L) (vs. physical therapy: RR = 1.36, 95% CI 1.16–1.59, SUCRA = 91.7%, low certainty; vs. Western medicine: RR = 1.36, 95% CI 1.15–1.59, SUCRA = 93.6%, low certainty) and for massage combined with acupuncture (M) (vs. physical therapy: RR = 1.35, 95% CI 1.13–1.62, SUCRA = 90.4%, low certainty; vs. Western medicine: RR = 1.35, 95% CI 1.13–1.61, SUCRA = 90.4%, low certainty).

Further comparisons among acupuncture-related interventions revealed that fire needling (J), warm acupuncture-moxibustion (D), moxibustion (K), acupuncture with cupping (F), electroacupuncture (B), and massage (C) were all significantly more effective than physical therapy and Western medicine (Appendix Table S10.1). Based on the CINeMA assessment, the certainty of evidence for total effectiveness was generally rated as low to moderate (Appendix Table S6.2).

### VAS pain scores

**Figure 5:**
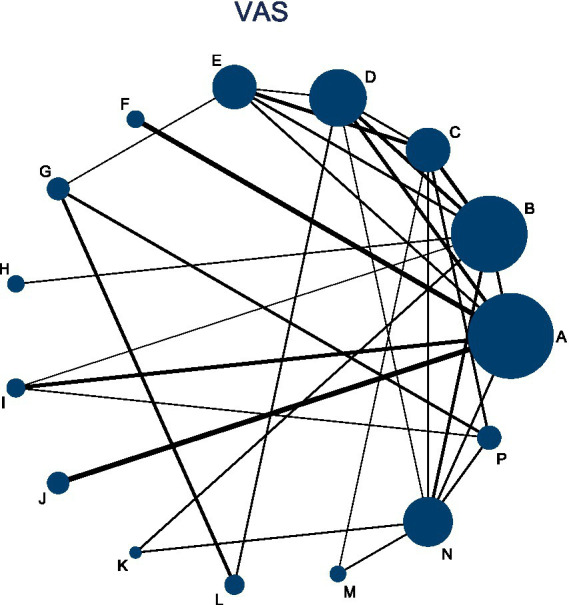
Network diagram of acupuncture and related therapies for VAS.

**Figure 6:**
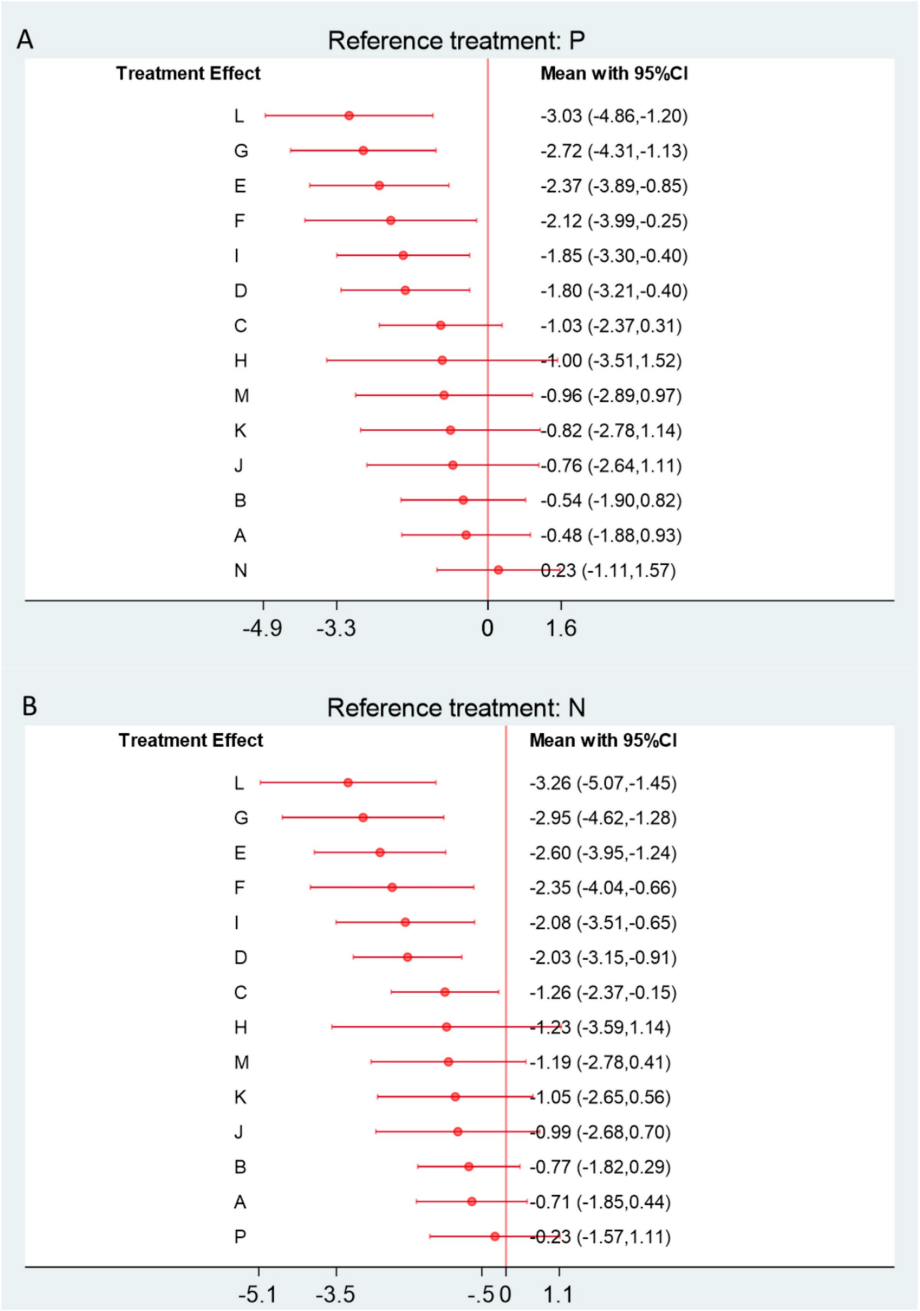
**(A)** Forest plot comparing acupuncture and related therapies with physical therapy for VAS outcomes. **(B)** Forest plot comparing acupuncture and related therapies with Western medicine for VAS outcomes. A, Acupuncture; B, Electroacupuncture; C, Massage; D, Warm Acupuncture-moxibustion; E, Small Needle Knife; F, Acupuncture and Cupping Therapy; G, Joint Mobilization; H, Bianshi Therapy; I, Floating Needle; J, Fire Meedling; K, Moxibustion; L, Joint Mobilization + Warm Acupuncture-Moxibustion; M, Massage+Acupuncture; N, Western Medicine; O, Acupoint Injection; P, Physical Therapy; Q, Functional Exercise.

**Figure 7:**
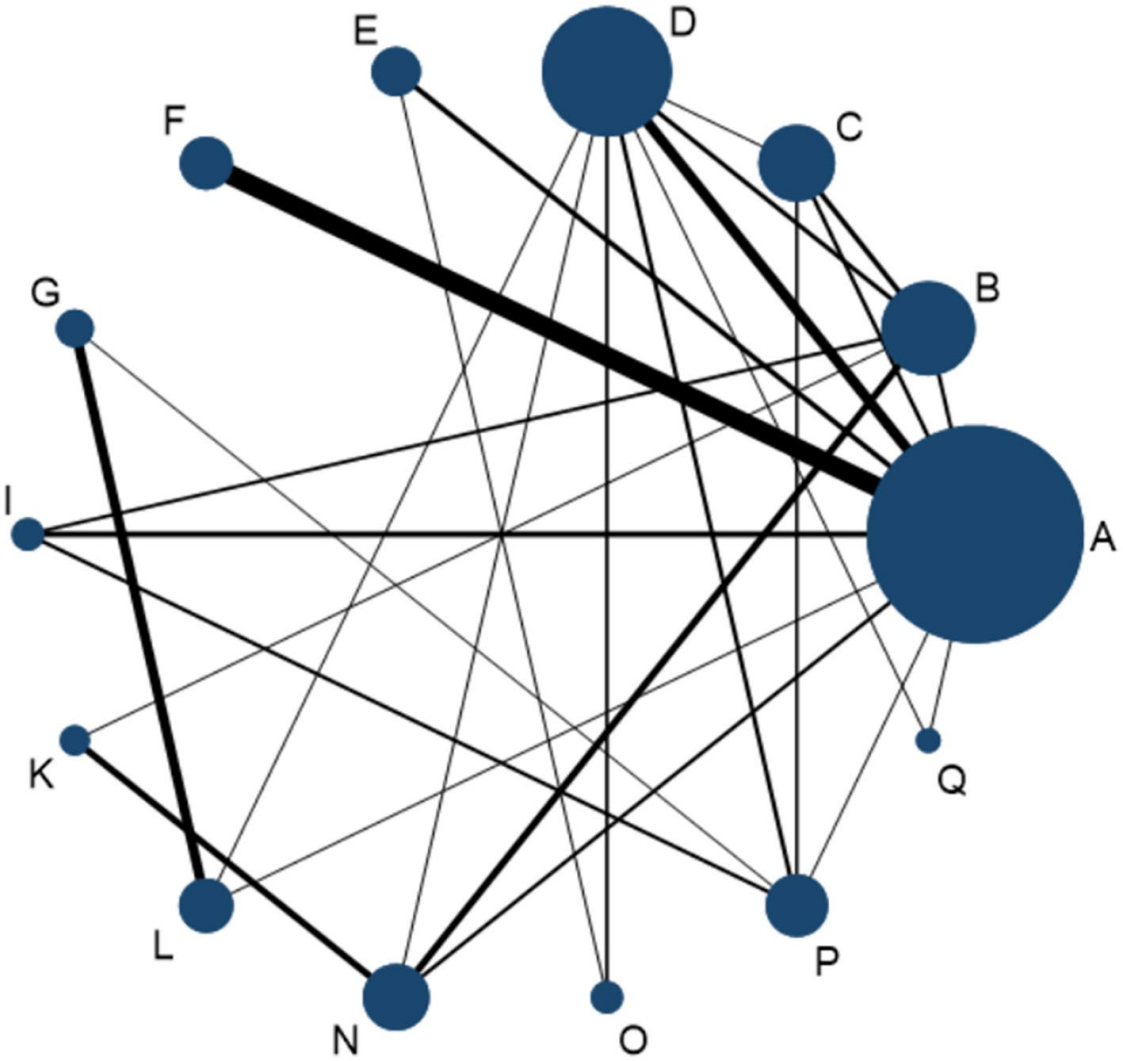
Network diagram comparing apparent healing rates among acupuncture-related therapies for frozen shoulder.

This analysis included 39 RCTs involving a total of 3,750 participants. The network diagram (Figure 5) illustrates the direct comparisons between acupuncture-related therapies and physical therapy. The thickness of the connecting lines represents the frequency of direct comparisons, with relatively more studies comparing Acupuncture (A), Floating Needle (I), Acupuncture and Cupping Therapy (F), and Moxibustion (K). The forest plot (Figure 6) shows that Western Medicine (N) tended to provide greater reductions in VAS pain scores compared with 13 other interventions, although seven therapies showed no significant advantage. Specifically, compared with Physical Therapy (P), Western Medicine demonstrated a mean difference (MD) of 0.23 (95% CI: −1.11 to 1.57; SUCRA = 92.4%), with low certainty of evidence. In contrast, Acupuncture (A), Electroacupuncture (B), Fire Needling (J), Moxibustion (K), Massage combined with Acupuncture (M), and Bianshi Therapy (H) did not differ significantly from Physical Therapy or Western Medicine in pain reduction. Further details on pairwise comparisons for VAS outcomes, along with SUCRA probabilities and related rankings, are presented in Appendix Table S10.2 (Figure 7).

### Apparent healing rate

**Figure 8:**
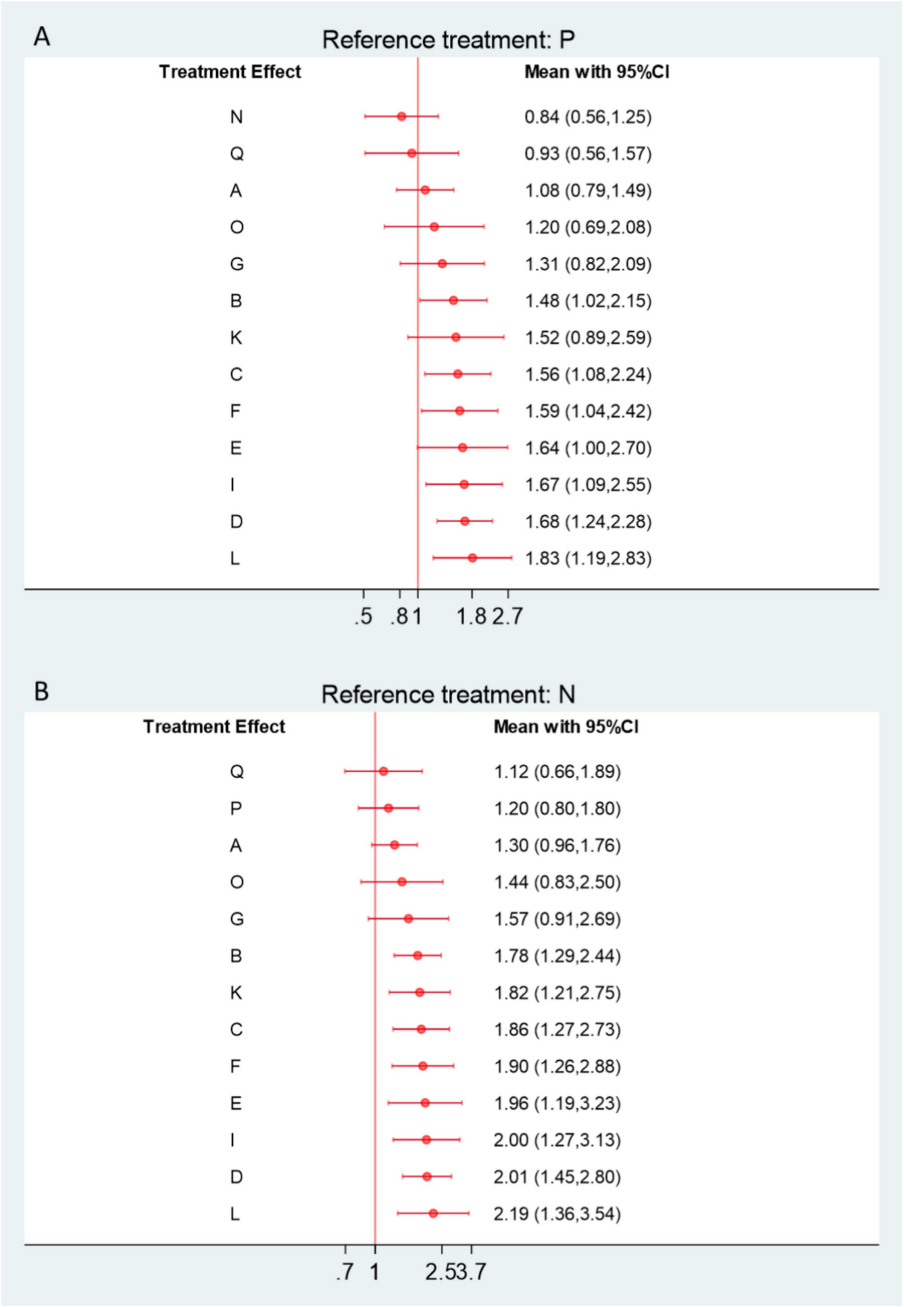
**(A)** Forest plot comparing apparent healing rates between acupuncture-related therapies and physical therapy controls. **(B)** Forest plot comparing apparent healing rates between acupuncture-related therapies and Western medicine controls. A, Acupuncture; B, Electroacupuncture; C, Massage; D, Warm Acupuncture-moxibustion; E, Small Needle Knife; F, Acupuncture and Cupping Therapy; G, Joint Mobilization; H, Bianshi Therapy; I, Floating Needle; J, Fire Meedling; K, Moxibustion; L, Joint Mobilization + Warm Acupuncture-Moxibustion; M, Massage+Acupuncture; N, Western Medicine; O, Acupoint Injection; P, Physical Therapy; Q, Functional Exercise.

**Figure 9:**
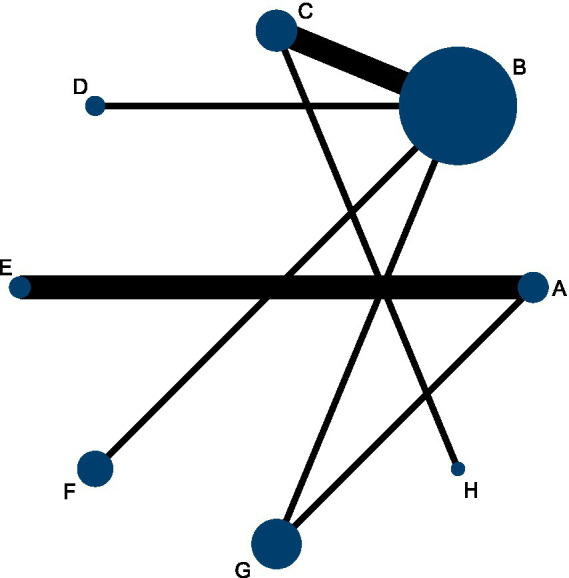
Network diagram of adverse reactions and withdrawal rates for acupuncture and related therapies in patients with frozen shoulder.

A total of 46 RCTs, involving 4,008 participants, were included in the network meta-analysis assessing apparent healing rates. As shown in Figure 8A, compared with physical therapy (P), 11 interventions demonstrated a trend toward improved apparent healing rates. Among them, Joint Mobilization + Warm Acupuncture-Moxibustion (L), Warm Acupuncture-Moxibustion (D), Floating Needle (I), Acupuncture and Cupping Therapy (F), Massage (C), and Electroacupuncture (B) achieved statistically significant improvements. When compared with Western medicine (N), 13 interventions performed better overall (Figure 8B), with eight showing statistically significant advantages. Notably, Joint Mobilization + Warm Acupuncture-Moxibustion (L) yielded the most pronounced effect (vs. P: RR = 1.83, 95% CI = 1.19–2.83, SUCRA = 83.3%, low certainty; vs. N: RR = 2.19, 95% CI = 1.36–3.54, SUCRA = 83.3%, low certainty). Warm Acupuncture-Moxibustion (D) and Floating Needle (I) also produced significant benefits: Warm Acupuncture-Moxibustion (vs. P: RR = 1.68, 95% CI = 1.24–2.28, SUCRA = 76.2%, high certainty; vs. N: RR = 2.01, 95% CI = 1.45–2.80, SUCRA = 76.2%, high certainty); Floating Needle (vs. P: RR = 1.67, 95% CI = 1.09–2.55, SUCRA = 73.3%, moderate certainty; vs. N: RR = 2.00, 95% CI = 1.27–3.13, SUCRA = 73.3%, low–moderate certainty). Further details on SUCRA rankings and comparative analyses are provided in Appendix Table S10.3 (Figure 9).

### Adverse reactions and shedding

**Figure 10:**
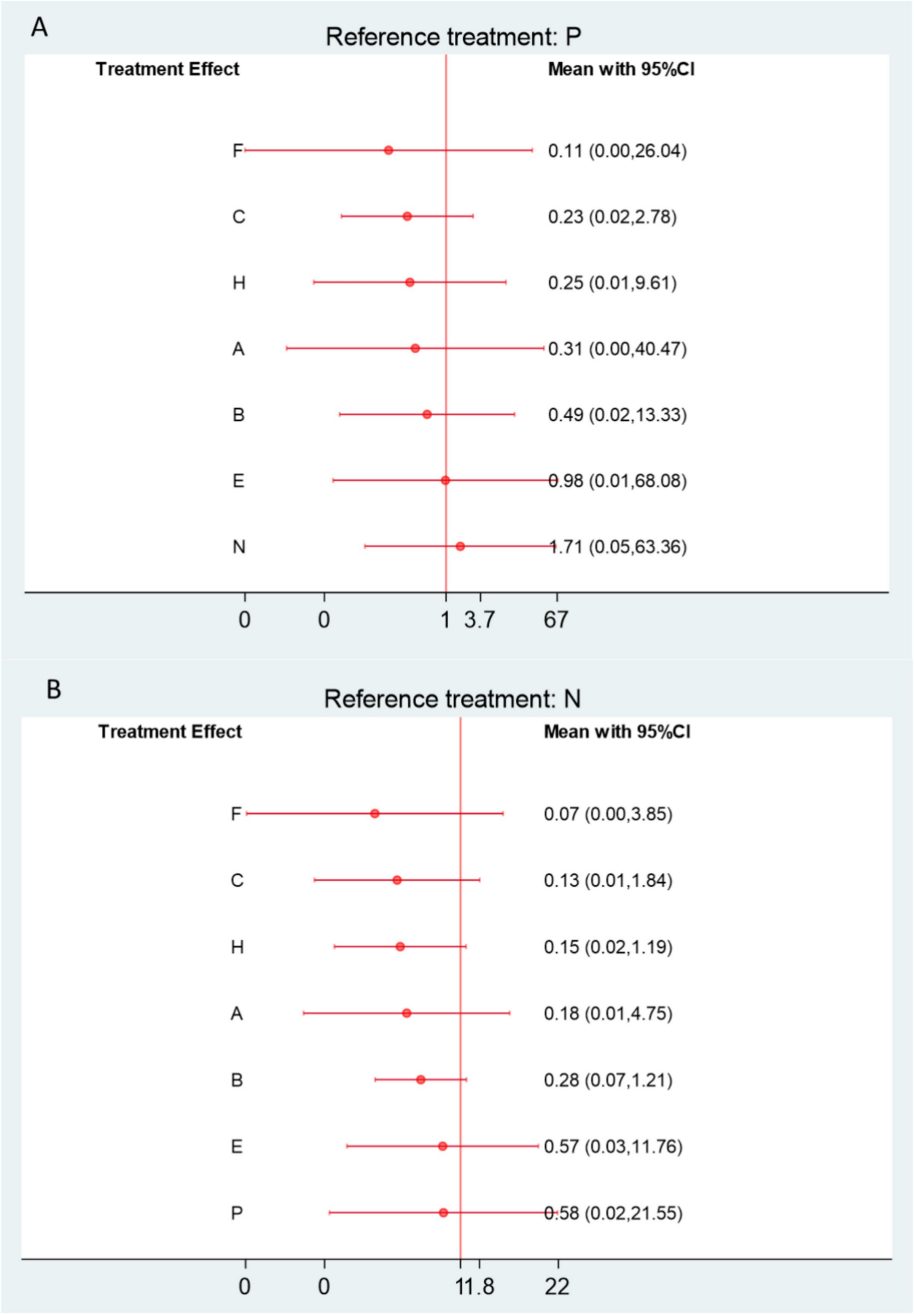
**(A)** Forest plot comparing adverse reactions and withdrawal rates between acupuncture-related therapies and physical therapy controls. **(B)** Forest plot comparing adverse reactions and withdrawal rates between acupuncture-related therapies and Western medicine controls. A, Acupuncture; B, Electroacupuncture; C, Massage; D, Warm Acupuncture-moxibustion; E, Small Needle Knife; F, Acupuncture and Cupping Therapy; G, Joint Mobilization; H, Bianshi Therapy; I, Floating Needle; J, Fire Meedling; K, Moxibustion; L, Joint Mobilization + Warm Acupuncture-Moxibustion; M, Massage+Acupuncture; N, Western Medicine; O, Acupoint Injection; P, Physical Therapy; Q, Functional Exercise.

A network meta-analysis (NMA) was conducted to evaluate adverse events associated with acupuncture and related therapies. Compared with physical therapy (P), interventions such as acupuncture and cupping therapy (F), massage (C), Bianshi therapy (H), acupuncture (A), and electroacupuncture (B) demonstrated a lower trend of adverse events, although these differences were not statistically significant (Figure 10A). When compared with Western medicine (N), seven interventions showed reduced relative risk (RR) of adverse events, again without significant differences (Figure 10B).

**Figure 11:**
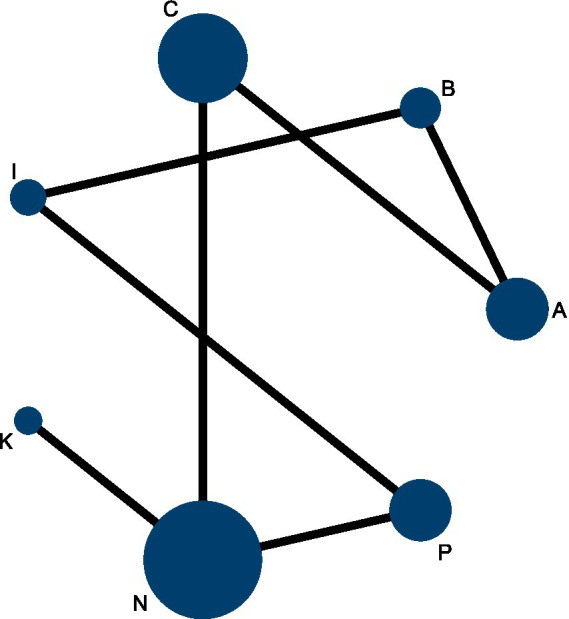
Network diagram of acupuncture and related therapies for frozen shoulder based on CMS scores.

Specifically, for acupuncture and cupping therapy (F), the RR was 0.11 (95% CI 0–26.04; SUCRA = 30%; low certainty) versus physical therapy, and 0.07 (95% CI 0–3.85; low certainty) versus Western medicine. For massage (C), the RR was 0.23 (95% CI 0.02–2.78; SUCRA = 35.2%; high certainty) compared with physical therapy, and 0.13 (95% CI 0.01–1.84; high certainty) versus Western medicine. Bianshi therapy (H) also showed a similar trend (RR = 0.25, 95% CI 0.01–9.61; SUCRA = 36.8% vs. P; RR = 0.15, 95% CI 0.02–1.19 vs. N) (Figure 11).

The most commonly reported adverse reactions included general swelling, transient hepatic enzyme elevation, local skin depigmentation, and localized muscle induration with urticaria. Other occasional events such as vasovagal fainting during acupuncture were also documented (Appendix 13). Notably, local depigmentation and muscle induration with urticaria occurred exclusively in the control groups, with an incidence of approximately 6%. Overall, adverse events were less frequent in the treatment groups than in the controls, though none of the differences reached statistical significance.

### CMS

**Figure12:**
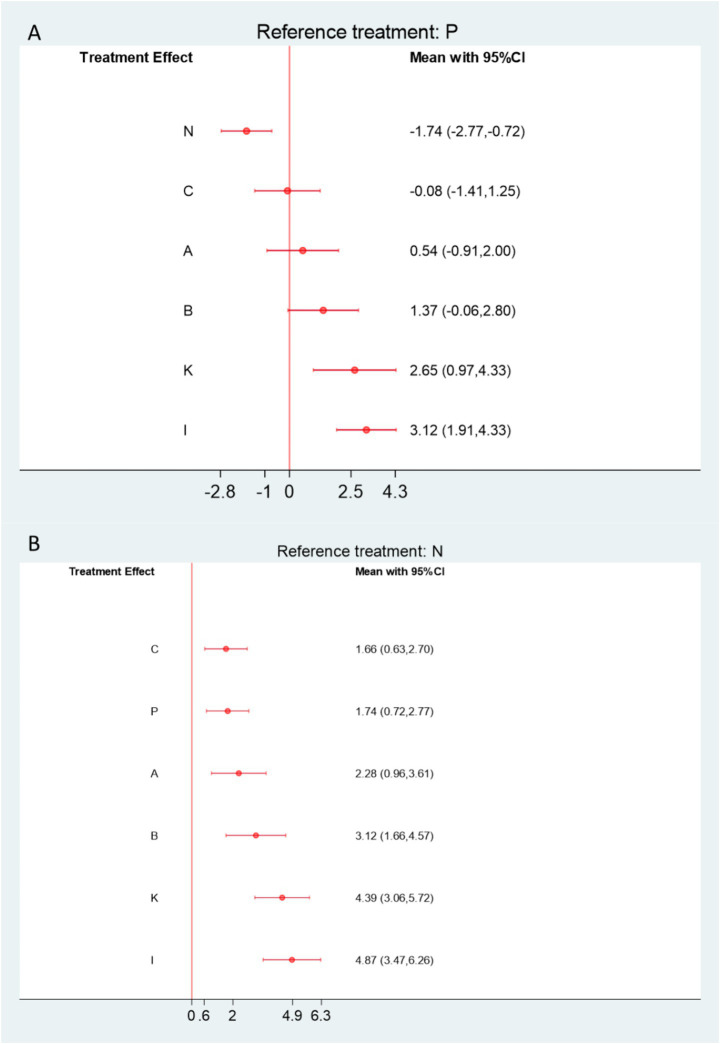
**(A)** Forest plot comparing acupuncture and related therapies with physical therapy controls for Constant–Murley Shoulder (CMS) scores. **(B)** Forest plot comparing acupuncture and related therapies with Western medicine controls for CMS scores. A, Acupuncture; B, Electroacupuncture; C, Massage; D, Warm Acupuncture-moxibustion; E, Small Needle Knife; F, Acupuncture and Cupping Therapy; G, Joint Mobilization; H, Bianshi Therapy; I, Floating Needle; J, Fire Meedling; K, Moxibustion; L, Joint Mobilization + Warm Acupuncture-Moxibustion; M, Massage+Acupuncture; N, Western Medicine; O, Acupoint Injection; P, Physical Therapy; Q, Functional Exercise.

The network meta-analysis (NMA) for the Constant–Murley Shoulder (CMS) score included seven randomized controlled trials comprising 1,116 participants. Among patients with frozen shoulder, four interventions demonstrated potential benefits over physical therapy, with Floating Needle (I) and Moxibustion (K) showing statistically significant improvements in CMS scores (Figure 12A). When compared with Western medicine, six interventions yielded superior CMS outcomes, all reaching statistical significance (Figure 12B).

Specifically, Floating Needle (I) significantly improved shoulder function compared with both controls (vs. Physical Therapy: SMD = 3.12, 95% CI = 1.91–4.33, SUCRA = 94.8%, moderate certainty; vs. Western Medicine: SMD = 4.87, 95% CI = 3.47–6.26, low certainty). Similarly, Moxibustion (K) demonstrated marked improvements (vs. Physical Therapy: SMD = 2.65, 95% CI = 0.97–4.33, SUCRA = 86.5%, low certainty; vs. Western Medicine: SMD = 4.39, 95% CI = 3.06–5.72, low certainty). Electroacupuncture (B) and Manual Acupuncture (A) also exhibited significant advantages over both control treatments. Additional SUCRA rankings and indirect comparison details are provided in Appendix Table S10.5.

### Calibrated comparison funnel plot

Comparative-corrected funnels for each of the included outcome metrics were plotted using Stata 18.0 software when there were more than 10 included studies, and the funnels were symmetrical, suggesting that there was a low likelihood of publication bias among the included studies (Appendix Figures S7.1–S7.3). The comparative-corrected funnels for VAS scores had a partial small-sample bias, but the funnels for the VAS scores, the overall effectiveness rate, and the Cure-Marked Improvement Rate were clearly symmetrical, suggesting that the current study is less likely to have publication bias or small-sample effects.

### Subgroup analysis

To explore potential clinical heterogeneity, a subgroup network meta-analysis was conducted according to treatment duration (<14 days, 14–28 days, and >28 days). For short-term treatment (≤14 days), joint mobilization combined with warm acupuncture-moxibustion (L), floating needle therapy (I), and fire needling (J) demonstrated greater short-term improvement in overall effectiveness compared with physical therapy (P) and Western medicine (N). However, these differences were not statistically significant when compared with small needle knife therapy (E) (Appendix Tables S13.1–S13.2). In the subgroup of moderate treatment duration (14–28 days), the ranking of overall effectiveness remained consistent with the primary analysis, with small needle knife therapy (E), joint mobilization plus warm acupuncture-moxibustion (L), and massage combined with acupuncture (M) showing the most favorable outcomes (Appendix Table S13.4). For long-term treatment (>28 days), massage combined with acupuncture (M), massage (C), and acupuncture (A) demonstrated significant superiority over physical therapy, suggesting that these interventions may provide more sustained therapeutic benefits with prolonged treatment. Overall, the subgroup results were consistent with the main network estimates, indicating that treatment duration was not a major source of clinical heterogeneity in the network.

## Discussion

This network meta-analysis provides a comprehensive comparison of acupuncture and related therapies for frozen shoulder, integrating evidence from 68 randomized controlled trials. Overall, acupuncture-based interventions demonstrated superior clinical effectiveness compared with both physical therapy and Western medicine, particularly for improving overall effectiveness rate and apparent healing rate. Among the evaluated therapies, small needle knife therapy, joint mobilization combined with warm acupuncture-moxibustion, and massage combined with acupuncture consistently ranked among the most effective approaches. Functional recovery, assessed by the Constant–Murley Shoulder score, was notably enhanced by floating needle and moxibustion therapies, while reductions in pain intensity (VAS) were comparable across most interventions. Adverse events were infrequent, generally mild, and occurred less often in treatment groups than in controls. Across all analyses, statistical heterogeneity was low to moderate, consistency between direct and indirect comparisons was maintained, and no significant publication bias was observed. The overall certainty of evidence was rated as low to moderate, largely reflecting incomplete reporting and variability in study quality.

Modern management of frozen shoulder spans pharmacologic, physical, and surgical options. Nonsteroidal anti-inflammatory drugs and intra-articular corticosteroid injections provide rapid pain relief and short-term functional gains, but benefits often wane after discontinuation and long-term efficacy is limited (96, 97). Intensive physical therapy and manipulation under anesthesia can improve range of motion, yet adherence, discomfort, and risks such as joint trauma or recurrent adhesions constrain their use (98). Arthroscopic capsular release is reserved for refractory disease and can be effective, albeit invasive and resource-intensive (99).

Against this backdrop, acupuncture and related therapies offer a minimally invasive alternative that bridges pharmacologic and physical strategies. Evidence from GRASP and other comparative trials suggests that acupuncture can achieve comparable—or superior—improvements in pain and mobility relative to NSAIDs or standard physical therapy, without rebound effects or procedure-related risks (100). Taken together, these data position acupuncture-based interventions as plausible complementary or stand-alone options, particularly for patients with chronic or treatment-resistant presentations where long-term safety and tolerability are paramount (96–100).

In fact, the mechanism of acupuncture and its related therapies for the treatment of diseases has been studied more extensively, mainly involving both anti-inflammatory and analgesic aspects. ① Inhibition of inflammatory factor release: Langevin (101) proposed that electroacupuncture stimulates fibroblasts to activate growth and showed that electroacupuncture stimulates the release of platelet-derived growth factor (PDGF), insulin-like growth factor 1 (IGF-1), and interleukin 6 (IL-6) as well as the inhibition of NF-KB DNA-binding activity in the ganglion of the monkey’s middle brain, which reduces the inflammatory response in tissue damage and accelerate the repair process of damaged tissues; Dingyu Zhu (102) demonstrated through experiments that electroacupuncture can effectively reduce the expression of inflammatory factors such as IL-1β and TNF-*α* and NLRP3 proteins, and alleviate inflammation. However, this conclusion still needs to be validated by more studies, especially regarding the elucidation of the specific mechanism of action of NLRP3 and the mechanism of analgesia, and the effectiveness in human patients needs to be further confirmed. ② Adjustment of neurotransmitter release: 5-HT (5-hydroxytryptamine) is a peripheral analgesic, Acupuncture and Thunder Fire Acupuncture can reduce its level, reducing inflammation and pain (103); PGE2 (prostaglandin E2) is analgesic in inflammation, circumferential stabbing method and Warm Acupuncture-Moxibustion can reduce its level, relieving pain in Frozen Shoulder (104); Bradykinin is involved in inflammatory pain and neuropathic pain, carpal and ankle acupuncture and beryllium acupuncture can reduce its level, improving microcirculation, relieving pain levels, improving microcirculation and relieving pain (105); in inhibiting the release of endogenous vasodilatory factors, NO (nitric oxide) exacerbates inflammatory responses, Warm Acupuncture-Moxibustion can down-regulate NO levels, reducing the release of inflammatory mediators and relieving pain (106). (iii) Modulation of central sensitization: central sensitization makes the nervous system hypersensitive to stimuli, leading to persistent pain. Acupuncture reduces nociceptive sensitization by increasing *β*-EP and decreasing SP, relieving chronic pain symptoms and reducing inflammatory exudation (107). All of these results confirm that Acupuncture and its related therapies can be a potential complementary treatment option when treating chronic Frozen Shoulder.

### Clinical implications

This study provides a comparative framework that may assist clinicians in selecting individualized acupuncture-based regimens for patients with frozen shoulder. The findings suggest that small needle knife therapy and combined approaches such as joint mobilization plus warm acupuncture-moxibustion or massage plus acupuncture could be prioritized for patients presenting with chronic stiffness or capsular contracture, where restoring joint mobility is the main therapeutic goal. In contrast, modalities such as warm acupuncture-moxibustion, moxibustion, or fire needling may be more appropriate during the painful or inflammatory stage, when rapid analgesia and anti-inflammatory effects are desirable.

From a clinical integration perspective, acupuncture-related therapies appear most beneficial when used as adjuncts to standard physical therapy or rehabilitation protocols rather than as standalone treatments. Their favorable safety profile and multidimensional mechanisms—targeting pain modulation, inflammation control, and soft-tissue remodeling—make them particularly suitable for patients with medication intolerance, contraindications to corticosteroid injections, or poor response to conventional physiotherapy.

Although subgroup analysis based on treatment duration provided preliminary insights into differential responses, further evidence is needed to clarify stage-specific benefits and long-term outcomes across diverse patient populations. Importantly, the current evidence supports a shift toward stage-specific and mechanism-based application of acupuncture in frozen shoulder management, rather than uniform use across all disease phases. In clinical practice, tailoring acupuncture modalities according to symptom duration, inflammatory status, and patient tolerance may enhance outcomes and reduce unnecessary interventions. Establishing such individualized protocols could bridge the existing gap between traditional empirical approaches and modern evidence-based rehabilitation strategies.

### Strengths and limitations

The strengths of this study are: (i) Despite the growing interest in Acupuncture for Frozen Shoulder, a comprehensive quantitative comparison of its various forms has been lacking. This study aims to fill this gap by employing a reticulated meta-analysis. (ii) reticulated Meta-analysis generates probability rankings (e.g., SUCRA values), which visually demonstrates the strengths and weaknesses of different therapies; and (iii) based on the different needs of different groups of people who suffer from periarthritis, this study provides clinical guidance for the best therapy.

While this study provides valuable evidence on the comparative effectiveness of acupuncture-related therapies for frozen shoulder, several limitations should be acknowledged. First, although 84 randomized controlled trials were included, the overall methodological quality was generally low. Only three studies explicitly reported allocation concealment, and blinding procedures were inconsistently applied, which may increase the risk of bias and contribute to heterogeneity in the pooled results. Second, most of the included studies were conducted in China, with limited representation from other regions. This geographic concentration of evidence may introduce selection and language bias, thereby limiting the generalizability of the findings to broader populations. Third, variations in reporting of follow-up duration, treatment course, and baseline participant characteristics across studies restricted our ability to perform detailed subgroup analyses. Future research should prioritize high-quality, multicenter, and internationally collaborative RCTs with standardized protocols to strengthen the evidence base.

## Conclusion

This network meta-analysis indicates that small needle knife therapy, joint mobilization combined with warm acupuncture-moxibustion, and massage combined with acupuncture appear to be among the most effective acupuncture-related interventions for improving overall effectiveness in patients with frozen shoulder. Floating needle and moxibustion therapies show particular benefits in restoring shoulder function, while acupuncture-based treatments are generally safe and well tolerated. However, the overall certainty of evidence is moderate to low, mainly due to methodological limitations and potential bias across the included trials. These findings should therefore be interpreted with caution. Future large-scale, multicenter randomized controlled trials with rigorous design and standardized reporting are needed to strengthen the evidence base.

## Data Availability

The original contributions presented in the study are included in the article/Supplementary material, further inquiries can be directed to the corresponding author/s.

## References

[1] Li D St Angelo JM Taqi M. Adhesive capsulitis (frozen shoulder) In: Li D, editor. Stat Pearls. Treasure Island, FL: Stat Pearls Publishing (2025) 30422550

[2] Patel R Urits I Wolf J Murthy A Cornett EM Jones MR . A comprehensive update of adhesive capsulitis and minimally invasive treatment options. Psychopharmacol Bull. (2020) 50:91–107. doi: 10.64719/pb.4384, PMID: 33633420 PMC7901130

[3] Ben-Arie E Kao PY Lee YC Ho WC Chou LW Liu HP. The effectiveness of acupuncture in the treatment of frozen shoulder: a systematic review and meta-analysis. Evid Based Complement Alternat Med. (2020) 2020:9790470. doi: 10.1155/2020/9790470, PMID: 33062030 PMC7532995

[4] Bouaicha S Wieser K Kriechling P Scholz-Odermatt SM. A large-scale assessment of the healthcare burden of adhesive capsulitis of the shoulder joint. Swiss Med Wkly. (2020) 150:w20188. doi: 10.4414/smw.2020.20188, PMID: 32083705

[5] Ewald A. Adhesive capsulitis: a review. Am Fam Physician. (2011) 83:417–22. PMID: 21322517

[6] De Sire A Agostini F Bernetti A Mangone M Ruggiero M Dinatale S . Non-surgical and rehabilitative interventions in patients with frozen shoulder: umbrella review of systematic reviews. J Pain Res. (2022) 15:2449–64. doi: 10.2147/JPR.S371513, PMID: 36016536 PMC9397530

[7] Rodgers S Brealey S Jefferson L McDaid C Maund E Hanchard N . Exploring the outcomes in studies of primary frozen shoulder: is there a need for a core outcome set? Qual Life Res. (2014) 23:2495–504. doi: 10.1007/s11136-014-0708-6, PMID: 24817317

[8] Koh KH. Corticosteroid injection for adhesive capsulitis in primary care: a systematic review of randomised clinical trials. Singapore Med J. (2016) 57:646–57. doi: 10.11622/smedj.2016146, PMID: 27570870 PMC5165171

[9] Li N Guo Y Gong Y Zhang Y Fan W Yao K . The anti-inflammatory actions and mechanisms of acupuncture from acupoint to target organs via neuro-immune regulation. J Inflamm Res. (2021) 14:7191–224. doi: 10.2147/JIR.S341581, PMID: 34992414 PMC8710088

[10] Asheghan M Aghda AK Hashemi E Hollisaz M. Investigation of the effectiveness of acupuncture in the treatment of frozen shoulder. Mater Sociomed. (2016) 28:253–7. doi: 10.5455/msm.2016.28.253-257, PMID: 27698596 PMC5034968

[11] Xu B Zhang L Zhao X Feng S Li J Xu Y. Efficacy of combining acupuncture and physical therapy for the Management of Patients with Frozen Shoulder: a systematic review and Meta-analysis. Pain Manag Nurs. (2024) 25:596–605. doi: 10.1016/j.pmn.2024.06.009, PMID: 38991907

[12] Wang X Hai X Jiang D Yin L Li H Wang Q . Efficacy and safety of warm needle treatment for scapulohumeral periarthritis: a protocol for systematic review and meta-analysis. Medicine (Baltimore). (2020) 99:e23237. doi: 10.1097/MD.0000000000023237, PMID: 33217841 PMC7676557

[13] Page MJ McKenzie JE Bossuyt PM Boutron I Hoffmann TC Mulrow CD . The PRISMA 2020 statement: an updated guideline for reporting systematic reviews. BMJ. (2021) 372:n71. doi: 10.1136/bmj.n71 33782057 PMC8005924

[14] Hutton B Salanti G Caldwell DM Chaimani A Schmid CH Cameron C . The PRISMA extension statement for reporting of systematic reviews incorporating network meta-analyses of health care interventions: checklist and explanations. Ann Intern Med. (2015) 162:777–84. doi: 10.7326/M14-2385, PMID: 26030634

[15] Hao Z Yanjun S Shiliang L. Clinical guidelines for Acupotomy in the treatment of periarthritis of the shoulder. China J Tradit Chin Med Pharm. (2024) 39:5989–94.

[16] Sterne JAC Savović J Page MJ Elbers RG Blencowe NS Boutron I . RoB 2: a revised tool for assessing risk of bias in randomised trials. BMJ. (2019) 366:l4898. doi: 10.1136/bmj.l4898, PMID: 31462531

[17] Nikolakopoulou A Higgins JPT Papakonstantinou T Chaimani A del Giovane C Egger M . CINeMA: an approach for assessing confidence in the results of a network meta-analysis. PLoS Med. (2020) 17:e1003082. doi: 10.1371/journal.pmed.1003082, PMID: 32243458 PMC7122720

[18] Papakonstantinou T Nikolakopoulou A Higgins JPT Egger M Salanti G. CINeMA: software for semiautomated assessment of the confidence in the results of network meta-analysis. Campbell Syst Rev. (2020) 16:e 1080. doi: 10.1002/cl2.1080, PMID: 37131978 PMC8356302

[19] White IR. Multivariate random-effects meta-regression: updates to mvmeta. Stata J. (2011) 11:255–70. doi: 10.1177/1536867X1101100206

[20] Rücker G. Network meta-analysis, electrical networks and graph theory. Res Synth Methods. (2012) 3:312–24. doi: 10.1002/jrsm.1058, PMID: 26053424

[21] Langan D Higgins JPT Jackson D Bowden J Veroniki AA Kontopantelis E . A comparison of heterogeneity variance estimators in simulated random-effects meta-analyses. Res Synth Methods. (2019) 10:83–98. doi: 10.1002/jrsm.1316, PMID: 30067315

[22] Turner RM Davey J Clarke MJ Thompson SG Higgins JP. Predicting the extent of heterogeneity in meta-analysis, using empirical data from the Cochrane database of systematic reviews. Int J Epidemiol. (2012) 41:818–27. doi: 10.1093/ije/dys041, PMID: 22461129 PMC3396310

[23] Chawla N Anothaisintawee T Charoenrungrueangchai K Thaipisuttikul P McKay GJ Attia J . Drug treatment for panic disorder with or without agoraphobia: systematic review and network meta-analysis of randomised controlled trials. BMJ. (2022) 376:e066084. doi: 10.1136/bmj-2021-066084, PMID: 35045991 PMC8767458

[24] Da Costa BR Juni P. Systematic reviews and meta-analyses of randomized trials: principles and pitfalls. Eur Heart J. (2014) 35:3336–45. doi: 10.1093/eurheartj/ehu424, PMID: 25416325

[25] Van Valkenhoef G Dias S Ades AE Welton NJ. Automated generation of node-splitting models for assessment of inconsistency in network meta-analysis. Res Synth Methods. (2016) 7:80–93. doi: 10.1002/jrsm.1167, PMID: 26461181 PMC5057346

[26] Bafeta A Trinquart L Seror R Ravaud P. Reporting of results from network meta-analyses: methodological systematic review. BMJ. (2014) 348:1741. doi: 10.1136/bmj.g1741, PMID: 24618053 PMC3949412

[27] Yang B Shi-ying J Xiaoqi Z. Clinical efficacy of small-needle knife with tuina in treating patients with periarthritis of the shoulder joint. China Med Guide South. (2020) 18:134–135+138. doi: 10.15912/j.cnki.gocm.2020.29.064

[28] Xingang B. Exploring the clinical effect of acupuncture and massage used in the treatment of periarthritis of the shoulder. Chin Foreign Womens Health Res. (2017) 15:37–9.

[29] Tao C Minlei Q Zhiping X . Observations on the efficacy of electroacupuncture at the shoulder point in the treatment of periarthritis of the shoulder joint. Shanghai J Acupunct Moxibustion. (2006) 1:21–2. doi: 10.13460/j.issn.1005-0957.2006.01.010

[30] Zhiwu C Hongping C. Evaluation of clinical efficacy of manipulation after needle knife overall release surgery for frozen shoulder. Acupunct Moxibustion Clin J. (2010) 26:1–3.

[31] Huizhen C Jing C Yunan L Chunyan Z . Clinical observation of warm acupuncture and moxibustion plus ultrashort wave in the treatment of frozen shoulder. Massage Guiding. (2006) 1:7–9.

[32] Libin C Qiuhua Y Xiuli NG. Clinical efficacy of silver needle combined with Lin's orthopedic massage on cervicogenic frozen shoulder. Inner Mongolia Tradit Chin Med. (2020) 39:144–5. doi: 10.16040/j.cnki.cn15-1101.2020.03.088

[33] Li C. Observation on the efficacy of warm needle with acupuncture exercise therapy in the treatment of 76 cases of frozen shoulder. Chin Med Clin Res. (2016) 8:120–6.

[34] Yingchun C Zhengen F Guoqi H. Randomized controlled clinical observation of warm acupuncture and moxibustion for the treatment of periarthritis of the shoulder (in English). J Acupunct Tuina Sci. (2015) 13:324–7.

[35] Yong C Xianmei L Zhuo C Kai W . Observation on the efficacy of 40 cases of frozen shoulder treated by exercise acupuncture with stabbing and cupping. New Chin Med. (2005) 5:60–1. doi: 10.13457/j.cnki.jncm.2005.05.033

[36] Yanxi D Peng W. Observations on 100 cases of frozen shoulder treated by small-needle knife relaxation with functional exercise. Chin Folk Ther. (2007) 4:50. doi: 10.19621/j.cnki.11-3555/r.2007.04.059

[37] Liqun D Mingbo Z. Efficacy analysis of shoulder three needles combined with tuina therapy in treating geriatric frozen shoulder patients. Inner Mongolia Tradit Chin Med. (2014) 33:50–1. doi: 10.16040/j.cnki.cn15-1101.2014.14.031

[38] Chunhai F. A randomized parallel-controlled study of warm acupuncture combined with microwave therapy for frozen shoulder. J Pract Chin Med Intern Med. (2015) 29:158–159+185. doi: 10.13729/j.issn.1671-7813.2015.09.69

[39] Jiagui F. Observation on the efficacy of needle knife treatment of frozen shoulder. J Pract Med Technol. (2007) 12:1618–9.

[40] Zhengen F Zeli C Chaofu D Lili W . Multicenter randomized controlled clinical study of fire acupuncture for the treatment of periarthritis of the shoulder joint. Shanghai J Acupunct Moxibustion. (2016) 35:707–9. doi: 10.13460/j.issn.1005-0957.2016.06.0707

[41] Chenglin Z Jing X Xing W Zhuqing Y . A multicenter randomized controlled clinical study on the treatment of early primary frozen shoulder with the combination of the retracting Tui na manipulation and discharging extracorporeal shock wave. Inner Mongolia Tradit Chin Med. (2024) 43:84–7. doi: 10.16040/j.cnki.cn15-1101.2024.02.031

[42] Jingyu G. Clinical observation on acupuncture treatment of frozen shoulder. Zhongguo Nankang Med. (2010) 22:410–3.

[43] Changqing G Na R Yangang L Yunxia L . A multicenter randomized controlled study of new stone therapy for frozen shoulder. Chin Acupunct Moxibustion. (2007) 1:633–7.

[44] Li H Min L Yu K Qingfeng L . A randomized controlled study of needle knife release combined with electroacupuncture in the treatment of frozen shoulder. Acupunct Moxibustion Clin J. (2019) 35:33–6.

[45] Yucai H. Observation on the clinical efficacy of floating needle therapy in the treatment of periarthritis of shoulder joint. Shenzhen J Integr Chin Western Med. (2019) 29:42–4. doi: 10.16458/j.cnki.1007-0893.2019.15.020

[46] Rongjuan H Zhiqiang Z Yuping D. Randomized parallel controlled study on the treatment of frozen shoulder by acupuncture, rapid stabbing with fire needle and triton method with small needle knife. J Pract Chin Med Intern Med. (2013) 27:117–9.

[47] Ping S Zhiping X Minlei Q Tao C . Clinical study on the treatment of periarthritis of the shoulder joint with electroacupuncture. Clin Study. (2006) 1:8–9+79.

[48] Shuirong H. Analgesic study of warm acupuncture with tuina in the treatment of frozen shoulder. Massage Guiding. (2008) 2:10–1.

[49] Lianxin H Xinzhou L Sxiaozhen N Suying Y . Clinical study on 30 cases of frozen shoulder treated by needle knife. Right River Medicine. (2013) 41:395–6.

[50] Zhao H. Randomized controlled clinical study of manual acupressure combined with acupuncture and cupping in the treatment of periarthritis of the shoulder. J Pract Chin Med. (2012) 26:96–9698.

[51] Jinguo J. Observation on the efficacy of warm acupuncture with tuina in treating acute-phase frozen shoulder. China Rural Health. (2019) 11:85–5.

[52] Decong K Hongjian L Jianfei C. Observation on the therapeutic effect of warm acupuncture and moxibustion, exercise therapy combined with joint loosening technique in the treatment of frozen shoulder. Chin Med Sci. (2018) 8:42–4.

[53] Peizheng L. A randomized parallel controlled study of massage therapy for periarthritis of the shoulder. J Pract Chin Med. (2013) 27:157–9.

[54] Junhe L Yunting S Yanwu W. Clinical effect of small needle knife with mulligan's dynamic joint release in the treatment of adhesive stage of frozen shoulder. China Med Herald. (2019) 16:159–62.

[55] Xinwei L Keping T Jia D Yafang S . Therapeutic observation of Fu's subcutaneous needling for scapulohumeral periarthritis. J Acupunct Tuina Sci. (2017) 15:281–4.

[56] Yan L Huagong L. Warm acupuncture and moxibustion in the treatment of 112 cases of frozen shoulder. J Tradit Chin Med. (2006) 4:38–9.

[57] Linyan L Kunming L Haini Y . Clinical efficacy observation of electro-acupuncture circumferential stabbing method in the treatment of acute-phase frozen shoulder. Chin Med Clin Res. (2019) 11:92–5.

[58] Xinxiao L Si C. *Electrothermal stone warming therapy for the treatment of wind-cold-damp type shoulder condensation randomized controlled recent clinical efficacy study*. Chinese acupuncture and moxibustion society stone and gua sha professional committee. Proceedings of the 2016 academic annual meeting of the Chinese acupuncture and moxibustion society stone and gua sha professional committee. Wangjing Hospital, China Academy of Traditional Chinese Medicine, (2016).

[59] Ziling L Xiaojun Z Jiaying L. Clinical study on the treatment of frozen shoulder by warm acupuncture combined with joint loosening. Chin Rehabil Theory Pract. (2011) 17:997–8.

[60] Jianwei L. Clinical observation on 80 cases of frozen shoulder in the elderly treated by tuina manipulation with acupuncture. J Guiyang Coll Tradit Chin Med. (2013) 35:307–8.

[61] Ming L Litao H. Clinical observation on 50 cases of frozen shoulder treated by warm needle combined with joint loosening. Pract Hosp Clin J. (2014) 11:136–8.

[62] Yan S Chunli D. Clinical efficacy observation of warm acupuncture with joint loosening in the treatment of frozen shoulder. Inner Mongolia Tradit Chin Med. (2017) 36:133–3.

[63] Yuanyuan L Zu A Yufeng X. Clinical observation on 30 cases of frozen shoulder treated by acupuncture with acupoint injection. Heilongjiang Tradit Chin Med. (2007) 36:39–40.

[64] Weiping L Xiaomin Y Xinlan C Jieyan Z . Warm acupuncture and moxibustion with exercise therapy in the treatment of periarthritis of the shoulder joint. Chin Rehabil Theory Pract. (2006) 2:154–5.

[65] Lihui M Haokai G Wei Z. Effects of warm acupuncture combined with ultrashort wave and dynamic interferential electricity on shoulder joint movement and pain in patients with frozen shoulder. Chin Med Inf. (2022) 39:55–9. doi: 10.19656/j.cnki.1002-2406.20220111

[66] Wei M. Nursing analysis of balanced fire cupping combined with thunder fire moxibustion in the treatment of shoulder condensation. China Health Stand Manag. (2018) 9:151–2.

[67] Shangxi P. A randomized parallel controlled study of warm acupuncture for frozen shoulder. J Pract Chin Med Intern Med. (2017) 31:60–2. doi: 10.13729/j.issn.1671-7813.2017.11.21

[68] Ye Q Long Q. Sea mud moxibustion paste combined with acupuncture in the treatment of frozen shoulder and its effect on shoulder joint function. China Tradit Chin Med Mod Distance Educ. (2019) 17:71–3.

[69] Si C Xinxiao L Zhenyu Z Yiping F . Randomized controlled clinical efficacy study of electrothermal stone warming therapy for the treatment of wind-cold-damp type shoulder condensation. Mod Chin Med Clin. (2016) 23:12–5.

[70] Hui S Jianqiao F Bangwei L Wenjie C *Evaluation of the efficacy of different acupuncture therapies for the treatment of frozen shoulder*. China Association of Acupuncture-Moxibustion. 2011 proceedings of the annual meeting of the China Association of Acupuncture and Moxibustion (abstract). The third hospital affiliated to Zhejiang University of traditional Chinese medicine; the first hospital affiliated to Wenzhou medical college; Ningbo Hospital of Traditional Chinese Medicine (2011).

[71] Biaomin Q Jinxiong L. Clinical observation on the treatment of frozen shoulder by acupuncture combined with joint loosening. Clin J Acupunct Moxibustion. (2006) 5:14–5.

[72] Chenyao W Jianqiao F Wenjie C. Therapeutic effect of “shoulder three needles” electro-acupuncture combined with warm needle on pre-adhesion wind-cold-damp type frozen shoulder. J Chin Tradit Med. (2011) 29:1063–5. doi: 10.13193/j.archtcm.2011.05.128.wangchy.010

[73] Fengchuan W. Clinical efficacy analysis of small-needle knife therapy in the treatment of recalcitrant frozen shoulder. Southwest Mil Med. (2008) 2:74–5.

[74] Hongwei W Maifang J. Treatment of 50 cases of frozen shoulder by acupuncture and canning with physiotherapy. Shaanxi Tradit Chin Med. (2006) 9:1125–6.

[75] Hongguo W. Clinical observation on the treatment of frozen shoulder with millifire needle combined with tendon manipulation. Shanxi Tradit Chin Med. (2022) 38:37–8. doi: 10.20002/j.issn.1000-7156.2022.09.013

[76] Hu W Hequn L Xinya BI Aili W . Clinical study on the treatment of frozen shoulder with wind-cold-damp paralysis by fire-acupuncture therapy. Acupunct Moxibustion Clin J. (2020) 36:39–42.

[77] Xi W Tong L Xiaoyin W. A randomized controlled trial study of floating needle therapy for the treatment of frozen shoulder. Massage Rehabil Med. (2018) 9:16–8. doi: 10.19787/j.issn.1008-1879.2018.23.007

[78] Bin W. Observation on the effect of acupuncture combined with active functional exercise staged treatment of frozen shoulder. China Med Guide. (2018) 16:177. doi: 10.15912/j.cnki.gocm.2018.02.149

[79] Chengju W Xin X Zhaojie X. Observation on the therapeutic effect of acupuncture and massage with Shenleng in the treatment of frozen shoulder. J Liaoning Univ Tradit Chin Med. (2007) 4:132–3. doi: 10.13194/j.jlunivtcm.2007.04.134.wuchj.087

[80] Guowei W. Observation on the efficacy of ultrasonic pulse electrical conduction combined with joint loosening in the treatment of frozen shoulder. Clin Med Eng. (2013) 20:6–7.

[81] Xianzhao L Jianxing D Kun Z Shuhang C . Clinical efficacy observation of acupuncture combined with meridian walking canning in the treatment of frozen shoulder. China Mod Drug Appl. (2023) 17:151–4. doi: 10.14164/j.cnki.cn11-5581/r.2023.23.040

[82] Xiangwei X. Clinical efficacy study of shoulder three needles combined with stabbing and cupping in the treatment of frozen shoulder. Contin Med Educ. (2020) 34:164–5.

[83] Kaisheng X Manwei H Liying Y . Clinical randomized controlled study of bamboo ring salt moxibustion for the treatment of frozen shoulder. Chin Acupunct Moxibustion. (2009) 29:77–80.

[84] Hongwei Y. Clinical research on the treatment of acute-phase frozen shoulder by tuina plus warm needle. Chin Med Clin Res. (2016) 8:109–10.

[85] Xuejun Y. A randomized parallel controlled study of acupuncture combined with tuina and functional forging for the treatment of frozen shoulder. J Pract Chin Med Intern Med. (2014) 28:137–9.

[86] Guanghao M Changqing G Foming Z. Clinical efficacy study and evaluation of acupuncture at the striated mouth point for the treatment of periarthritis of the shoulder joint. Shanghai J Acupunct Moxibustion. (2006) 9:23–4. doi: 10.13460/j.issn.1005-0957.2006.09.013

[87] Liang Z. Exploration of the mechanism of combining bee acupuncture with needle knife in the treatment of frozen shoulder and its clinical comparative study. Chin Med Clin Res. (2018) 10:76–7.

[88] Ruilian Z. Clinical observation on the treatment of periarthritis of shoulder joint with needle knife and manipulative release under brachial plexus anesthesia. China Mod Drug Appl. (2012) 6:63–4. doi: 10.3969/j.issn.1673-9523.2012.04.054

[89] Minming Z. Warm acupuncture and moxibustion combined with shoulder joint release in periarthritis of the shoulder. J Liaoning Univ Tradit Chin Med. (2017) 19:194–6. doi: 10.13194/j.issn.1673-842x.2017.04.059

[90] Tian Z Qing H Feng W Pengfei G . Acupuncture and cupping in the shoulder area combined with conventional acupuncture for frozen stage of frozen shoulder: a randomized controlled trial. Chin Acupunct. (2023) 43:911–5. doi: 10.13703/j.0255-2930.20221118-k0004 37577887

[91] Xiaoping Z. Study on the analgesic effect of warm acupuncture on frozen shoulder. J Pract Med. (2007) 1:127–8.

[92] Wang H Fan D Lan Q Gong H. Clinical Efficacy of Adhesion Release Under Brachial Plexus Block Plus Silver Needle Warm Acupuncture on Frozen Shoulder and Recovery of Limb Function. Altern Ther Health Med. (2025) 31:251–7. PMID: 38639609

[93] Wang BB Luo HP Yang XQ Huang XS. Herbal cake separated moxibustion combined with umbrella shaped acupuncture with round sharp needle for chronic scapulohumeral periarthritis of cold-damp stagnation. Zhongguo Zhen Jiu. (2020) 40:1291–4. doi: 10.13703/j.0255-2930.20190822-k0006, PMID: 33415870

[94] Lu J Sun JH Fu ZH Yuan JH Li J Ji AQ. Transient therapeutic effect and safety of superficial needling therapy for treatment of periarthritis of shoulder. Zhongguo Zhen Jiu. (2008) 28:414–6. PMID: 18630538

[95] Chen MY Pu QQ Liu SY Jiang ZY. Efficacy comparison of different stimulation therapies for periarthritis of shoulder. Zhongguo Zhen Jiu. (2013) 33:109–12. PMID: 23620934

[96] Buchbinder R Hoving JL Green S Hall S Forbes A Nash P. Short course prednisolone for adhesive capsulitis (frozen shoulder or stiff painful shoulder): a randomised, double blind, placebo controlled trial. Ann Rheum Dis. (2004) 63:1460–9. doi: 10.1136/ard.2003.018218, PMID: 15479896 PMC1754804

[97] Carette S Moffet H Tardif J Bessette L Morin F Frémont P . Intraarticular corticosteroids, supervised physiotherapy, or a combination of the two in the treatment of adhesive capsulitis of the shoulder: a placebo-controlled trial. Arthritis Rheum. (2003) 48:829–38. doi: 10.1002/art.10954, PMID: 12632439

[98] Kivimäki J Pohjolainen T Malmivaara A Kannisto M Guillaume J Seitsalo S . Manipulation under anesthesia with home exercises versus home exercises alone in the treatment of frozen shoulder: a randomized, controlled trial with 125 patients. J Shoulder Elb Surg. (2007) 16:722–6. doi: 10.1016/j.jse.2007.02.125, PMID: 17931902

[99] Pollock RG Duralde XA Flatow EL Bigliani LU. The use of arthroscopy in the treatment of resistant frozen shoulder. Clin Orthop Relat Res. (1994) 304:30–6. PMID: 8020231

[100] Molsberger AF Schneider T Gotthardt H Drabik A. German randomized acupuncture trial for chronic shoulder pain (GRASP) -a pragmatic, controlled, patientblinded, multi-Centre trial in an outpatient care environment. Pain. (2010) 151:146–54. doi: 10.1016/j.pain.2010.06.036, PMID: 20655660

[101] Langevin HM Bouffard NA Badger GJ Churchill DL Howe AK. Subcutaneous tissue fibroblast cytoskeletal remodeling induced by acupuncture: evidencefor a mechanotransduction-based mechanism. J Cell Physiol. (2006) 207:767–74. doi: 10.1002/jcp.20623, PMID: 16511830

[102] Dingyu Z. Clinical and basic research on inhibition of inflammation-mediated cartilage degeneration in osteoarthritis by electroacupuncture. Fuzhou: Fujian University of Traditional Chinese Medicine (2019).

[103] Wenjing Y. Effects of meridian stabbing combined with thunder fire moxibustion on serum 5-HT and SP levels in patients with adhesive stage frozen shoulder. Electron J Mod Med Health Res. (2020) 4:66–7.

[104] Fang W Yuanmian X Jiaquan L . Effects of perimortem stabbing method with warm acupuncture treatment on patients with frozen shoulder. Sichuan Tradit Chin Med. (2022) 40:194–7.

[105] Xi Z Sufang W Xiuxin LR. Clinical observation of wrist and ankle acupuncture combined with acupoint massage in the treatment of frozen shoulder. Hainan Med. (2020) 31:1425–8.

[106] Hongxin W Yanhui L Yunfeng L . Distribution of neuronal-type nitric oxide synthase-positive nerve fibers in the shoulder joint capsule of normal subjects. J Clin Exp Med. (2006) 9:1272–4.

[107] Weiwei W Manping S Yingying L. Clinical efficacy of gui zhi plus huang qi tang plus flavor combined with acupuncture on patients with wind-cold-damp type frozen shoulder. Chin Patent Med. (2020) 42:816–8.

