# Supplementary material for: Comparative effectiveness of acupuncture-related therapies for frozen shoulder: a systematic review and network meta-analysis
Source: Front Med (Lausanne). 2025 Nov 26;12:1673193. doi: 10.3389/fmed.2025.1673193 (PMC12689596; doi:10.3389/fmed.2025.1673193)
Supplement: Supplementary file 1 [file Data_Sheet_1.docx]

Supplementary Appendix

Comparative Effectiveness of Acupuncture and Related Therapies for

Frozen Shoulder:Systematic Review and Network Meta-Analysis

Rongyao Ji

Appendix

Appendix1: PRISMA-NMA list.......................................................................................................1

Appendix2: Search Strategy............................................................................................................ 5

Appendix3: Characteristics of Studies Included in the Literature..................................................7

Appendix4: Risk of Bias.....................................................................................13

Appendix 5:Assessment of Consistency and Inconsistency...........................................................15

Appendix6: Assessment of CINeMA....................................................................................19

Appendix7: Funnel Plot..................................................................................................................25

Appendix8: Network Maps.............................................................................................27

Appendix9: Forest Maps..................................................................................................30

Appendix10: SUCRA......................................................................................34

Appendix11：League Chart..............................................................................39

Appendix 1: PRISMA NMA Checklist

| **Section/Topic** | **Item #** | **Checklist Item** | **Reported on Page #** |
| --- | --- | --- | --- |
| **TITLE** |  |  |  |
| Title | 1 | This report is identified as a network-meta analysis | ***Title*** |
|  |  |  |  |
| **ABSTRACT** |  |  |  |
| Structured summary | 2 | Provide a structured summary including, as applicable:  **Background:** main objectives  **Methods:** data sources; study eligibility criteria, participants, and interventions; study appraisal; and *synthesis methods, such as network meta-analysis.*  **Results:** number of studies and participants identified; summary estimates with corresponding confidence/credible intervals; *treatment rankings may also be discussed. Authors may choose to summarize pairwise comparisons against a chosen treatment included in their analyses for brevity.*  **Discussion/Conclusions:** limitations; conclusions and implications of findings.  **Other:** primary source of funding; systematic review registration number with registry name. | Abstract |
|  |  |  |  |
| **INTRODUCTION** |  |  |  |
| Rationale | 3 | Describe the rationale for the review in the context of what is already known*, including mention of why a network meta-analysis has been conducted.* | **Introduction** |
| Objectives | 4 | Provide an explicit statement of questions being addressed, with reference to participants, interventions, comparisons, outcomes, and study design (PICOS). | **Introduction** |
|  |  |  |  |
| **METHODS** |  |  |  |
| Protocol and registration | 5 | Indicate whether a review protocol exists and if and where it can be accessed (e.g., Web address); and, if available, provide registration information, including registration number. | **METHODS** |
| Eligibility criteria | 6 | Specify study characteristics (e.g., PICOS, length of follow-up) and report characteristics (e.g., years considered, language, publication status) used as criteria for eligibility, giving rationale. *Clearly describe eligible treatments included in the treatment network, and note whether any have been clustered or merged into the same node (with justification).* | **Eligibility criteria** |
| Information sources | 7 | Describe all information sources (e.g., databases with dates of coverage, contact with study authors to identify additional studies) in the search and date last searched. | **Search strategy** |
| Search | 8 | Present full electronic search strategy for at least one database, including any limits used, such that it could be repeated. | **Search strategy** |
| Study selection | 9 | State the process for selecting studies (i.e., screening, eligibility, included in systematic review, and, if applicable, included in the meta-analysis). | **Search strategy** |
| Data collection process | 10 | Describe method of data extraction from reports (e.g., piloted forms, independently, in duplicate) and any processes for obtaining and confirming data from investigators. | **Data Extraction** |
| Data items | 11 | List and define all variables for which data were sought (e.g., PICOS, funding sources) and any assumptions and simplifications made. | **Data Extraction** |
| **Geometry of the network** | **S1** | Describe methods used to explore the geometry of the treatment network under study and potential biases related to it. This should include how the evidence base has been graphically summarized for presentation, and what characteristics were compiled and used to describe the evidence base to readers. | ***Appendix 8: Network maps*** |
| Risk of bias within individual studies | 12 | Describe methods used for assessing risk of bias of individual studies (including specification of whether this was done at the study or outcome level), and how this information is to be used in any data synthesis. | **Risk of Bias and Certainty of Evidence** |
| Summary measures | 13 | State the principal summary measures (e.g., risk ratio, difference in means). *Also describe the use of additional summary measures assessed, such as treatment rankings and surface under the cumulative ranking curve (SUCRA) values, as well as modified approaches used to present summary findings from meta-analyses.* | **Statistical analysis** |
| Planned methods of analysis | 14 | Describe the methods of handling data and combining results of studies for each network meta-analysis. This should include, but not be limited to:   - *Handling of multi-arm trials;* - *Selection of variance structure;* - *Selection of prior distributions in Bayesian analyses; and* - *Assessment of model fit.* | **Statistical analysis** |
| **Assessment of Inconsistency** | **S2** | Describe the statistical methods used to evaluate the agreement of direct and indirect evidence in the treatment network(s) studied. Describe efforts taken to address its presence when found. | Appendix 5: Evaluation of inconsistency and heterogeneity |
| Risk of bias across studies | 15 | Specify any assessment of risk of bias that may affect the cumulative evidence (e.g., publication bias, selective reporting within studies). | **Risk of bias, certainty of evidence, and consistency** |
| Additional analyses | 16 | Describe methods of additional analyses if done, indicating which were pre-specified. This may include, but not be limited to, the following:   - Sensitivity or subgroup analyses; - Meta-regression analyses; - *Alternative formulations of the treatment network; and* - *Use of alternative prior distributions for Bayesian analyses (if applicable).* | Subgroup Analysis |
| **RESULTS†** |  |  |  |
| Study selection | 17 | Give numbers of studies screened, assessed for eligibility, and included in the review, with reasons for exclusions at each stage, ideally with a flow diagram. | **Study selection** |
| **Presentation of network structure** | **S3** | Provide a network graph of the included studies to enable visualization of the geometry of the treatment network. | ***Appendix 8: Network maps*** |
| **Summary of network geometry** | **S4** | Provide a brief overview of characteristics of the treatment network. This may include commentary on the abundance of trials and randomized patients for the different interventions and pairwise comparisons in the network, gaps of evidence in the treatment network, and potential biases reflected by the network structure. | ***Appendix 8: Network maps*** |
| Study characteristics | 18 | For each study, present characteristics for which data were extracted (e.g., study size, PICOS, follow-up period) and provide the citations. | Characteristics of included studies |
| Risk of bias within studies | 19 | Present data on risk of bias of each study and, if available, any outcome level assessment. | Risk of bias, certainty of evidence, and consistency |
| Results of individual studies | 20 | For all outcomes considered (benefits or harms), present, for each study: 1) simple summary data for each intervention group, and 2) effect estimates and confidence intervals. *Modified approaches may be needed to deal with information from larger networks.* | ***Overall effectiveness rate*** |
| Synthesis of results | 21 | Present results of each meta-analysis done, including confidence/credible intervals. *In larger networks, authors may focus on comparisons versus a particular comparator (e.g. placebo or standard care), with full findings presented in an appendix. League tables and forest plots may be considered to summarize pairwise comparisons.* If additional summary measures were explored (such as treatment rankings), these should also be presented. | Appendix 9： Forest plot of the outcomes |
| **Exploration for inconsistency** | **S5** | Describe results from investigations of inconsistency. This may include such information as measures of model fit to compare consistency and inconsistency models, *P* values from statistical tests, or summary of inconsistency estimates from different parts of the treatment network. | Appendix 5: Evaluation of inconsistency and heterogeneity |
| Risk of bias across studies | 22 | Present results of any assessment of risk of bias across studies for the evidence base being studied. | Appendix6: CINeMA Assessment |
| Results of additional analyses | 23 | Give results of additional analyses, if done (e.g., sensitivity or subgroup analyses, meta-regression analyses*, alternative network geometries studied, alternative choice of prior distributions for Bayesian analyses,* and so forth). | Subgroup Analysis |
| **DISCUSSION** |  |  |  |
| Summary of evidence | 24 | Summarize the main findings, including the strength of evidence for each main outcome; consider their relevance to key groups (e.g., healthcare providers, users, and policy-makers). | Conclusions |
| Limitations | 25 | Discuss limitations at study and outcome level (e.g., risk of bias), and at review level (e.g., incomplete retrieval of identified research, reporting bias). *Comment on the validity of the assumptions, such as transitivity and consistency. Comment on any concerns regarding network geometry (e.g., avoidance of certain comparisons).* | **Strengths and Limitations** |
| Conclusions | 26 | Provide a general interpretation of the results in the context of other evidence, and implications for future research. | Conclusions |
| **FUNDING** |  |  |  |
| Funding | 27 | Describe sources of funding for the systematic review and other support (e.g., supply of data); role of funders for the systematic review. This should also include information regarding whether funding has been received from manufacturers of treatments in the network and/or whether some of the authors are content experts with professional conflicts of interest that could affect use of treatments in the network. | Funding |

**Appendix 2: Search Strategy**

**Table S1.**CNKI

| # |  |
| --- | --- |
| 1 | (Topics: Frozen shoulder) OR (Topics: Frozen shoulder) OR (Topics: Fifty shoulder) OR (Topics: Shoulder condensation) OR (Topics: Leaky shoulder) OR (Topics: Periarthritis) OR (Topics: Shoulder pain) OR (Topics: Adhesive shoulder arthritis) OR (Topics: Periarthritis of the shoulder) OR (Topics: Shoulder condensation) |
| 2 | (Topic: Acupuncture) OR (Topic: Warm Acupuncture) OR (Topic: Electroacupuncture) OR (Topic: Moxibustion) OR (Topic: Moxibustion) OR (Topic: Acupoint Embedding) OR (Topic: Fire Acupuncture) OR (Topic: Plum Blossom Needle Knocking) OR (Topic: Blood Pricking) |
| 3 | (Topic: RCT) OR (Topic: Randomized Controlled Trial) OR (Topic: rct) OR (Topic: Randomized Controlled Trial) OR (Topic: Randomized Controlled Study) |
| 4 | #1 AND #12 AND #3 |
|  |  |

**Table** S2.Wan Fang

| # |  |
| --- | --- |
| 1 | Acupuncture OR Warm Acupuncture OR Electroacupuncture OR Moxibustion OR Moxibustion OR Acupuncture Embedding OR Fire Needle OR Plum Blossom Needle Knocking OR Blood Stabbing |
| 2 | Frozen Shoulder OR Frozen Shoulder OR Fifty Shoulders OR Shoulder Condensation OR Leaky Shoulder OR Periarthritis OR Shoulder Pain OR Adhesive Shoulder Arthritis OR Periarthritis of the Shoulder OR Shoulder Condensation |
| 3 | RCT OR randomized controlled trial OR rct OR randomized controlled trial OR randomized controlled study |
| 4 | #1 AND #2AND #3 |

**Table** S3.VIP

| # |  |
| --- | --- |
| 1 | (((( title or keyword=Acupuncture OR title or keyword=Warm Acupuncture) OR title or keyword=Electric Acupuncture)  OR (Title or Keyword=Moxibustion OR Title or Keyword=Moxibustion)) OR (Title or Keyword=Physical Therapy  OR (title or keyword=Physical therapy)) |
| 2 | ((((((((( title or keyword=frozen shoulder OR title or keyword=frozen shoulder) OR title or keyword=frozen shoulder) OR title or keyword=fifty shoulder) OR title or keyword=Shoulder condensation) OR title or keyword=Shoulder pain) OR title or keyword=Shoulder pain) OR title or keyword=Shoulder pain) OR title or keyword=Arthritis periarthritis)  OR title or keyword=Shoulder pain) OR title or keyword=Adhesive shoulder arthritis) OR title or keyword=Shoulder periarthritis) OR title or keyword=Periarthritis OR title or keyword = periarthritis of shoulder) OR title or keyword = shoulder coagulopathy) |
| 3 | (((title or keyword=randomized controlled trial OR title or keyword=rct) OR title or keyword=randomized controlled trial OR title or keyword=randomized controlled study) OR title or keyword=randomized controlled study)) |
| 4 | #1 AND #2AND #3 |

**Table** S4. CBM

| *#* |  | |  |
| --- | --- | --- | --- |
| *1* | | “Moxibustion"[Common Fields:Intelligent] OR ‘Moxibustion’[Common Fields:Intelligent] OR ‘Blood Prick’[Common Fields:Intelligent] OR ”Plum Blossom Needle Percussion"[Common Fields:Intelligent] OR ‘Fire Needle’[Common Fields:Intelligent] OR ‘Electroacupuncture’[Common Fields:Intelligent] OR “Warm Acupuncture and Moxibustion”[Common Fields:Intelligent] OR “Acupuncture and Moxibustion”[Common Fields:Intelligent] | |
| *2* | “frozen shoulder” [Common Fields:Intelligent] OR ‘frozen shoulder’ [Common Fields:Intelligent] OR ‘frozen shoulder’ [Common Fields:Intelligent] OR ‘fifty shoulder’ [Common Fields:Intelligent] OR ‘shoulder condensation’ [Common Fields:Intelligent] OR ‘leaking shoulder’ [Common Fields:Intelligent] OR ‘periarticular arthritis’ [Common Fields:Intelligent] OR ‘shoulder joint pain’ [Common Fields:Intelligent] OR ‘adhesive shoulder arthritis’ [Common Fields. [Smart] OR “Periarthritis of shoulder”[Common Fields:Smart] | |  |
| *3* | “rct"[common field:smart] OR ‘randomized controlled trial’[common field:smart] OR ‘rct’[common field:smart] OR ‘randomized controlled trial’[common field:smart] OR ‘randomized controlled study’[common field:smart] OR ‘randomized controlled study’[common field:smart] | |  |
| *4* | #1 AND #2AND #3 | |  |

**Table** S5. PUBMED

| *#* |  |
| --- | --- |
| *1* | periarthritis humeroscapularis OR periarthritis of shoulder OR periarthritis of shoulder joint OR Frozen Shoulder OR shoulder periarthritis |
| *2* | Acupuncture OR acupuncture OR Acupuncture therapy OR Acupuncture-moxibustion therapy OR Moxibustion OR moxibustion OR moxibustion therapy OR therapy of moxibustion OR Fire Needle OR fire needle OR Plum Blossom Needle |
| *3* | clinicaltrial[Filter] OR randomizedcontrolledtrial[Filter] OR RCT) OR Randomized Controlled Trial OR rct OR Randomized Controlled Trial OR Randomized Controlled Study |
| *4* | #1 AND #2AND #3 |

**Table** S6. Cochrance

| *#* |  |
| --- | --- |
| *1* | periarthritis humeroscapularis OR periarthritis of shoulder OR periarthritis of shoulder joint OR Frozen Shoulder OR shoulder periarthritis |
| *2* | Acupuncture OR acupuncture OR Acupuncture therapy OR Acupuncture-moxibustion therapy |
| *3* | Moxibustion OR moxibustion OR moxibustion therapy OR therapy of moxibustion |
| *4* | Fire Needle OR fire needle OR fire-needle |
| *5* | Plum Blossom Needle |
| *6* | RCT OR rct OR Randomized Controlled Trial OR Randomized Controlled Trial OR Randomized Controlled Study |
| *7* | #1 AND #2 |
| *8* | #1 AND #3 |
| *9* | #1 AND #4 |
| *10* | #1 AND #5 |
| *11* | #7 OR #8 OR #9 OR #10 |
| *12* | #11 AND #6 |

**Table** S7. Web of science

| *#* |  |
| --- | --- |
| *1* | Frozen Shoulder (Topic) OR periarthritis humeroscapularis (Topic) OR periarthritis of shoulder (Topic) OR periarthritis of shoulder joint (Topic) OR shoulder periarthritis (Topic) and Preprint Citation Index (Exclude – Database) and Research Commons (Exclude – Database) |
| *2* | Acupuncture (Topic) OR acupuncture (Topic) OR Acupuncture therapy (Topic) OR Acupuncture-moxibustion therapy (Topic) and Preprint Citation Index (Exclude – Database) and Research Commons (Exclude – Database) |
| *3* | Moxibustion (Topic) OR moxibustion (Topic) OR moxibustion therapy (Topic) OR therapy of moxibustion (Topic) and Preprint Citation Index (Exclude – Database) and Research Commons (Exclude – Database) |
| *4* | Fire Needle (Topic) OR fire needle (Topic) OR fire-needle (Topic) and Preprint Citation Index (Exclude – Database) and Research Commons (Exclude – Database) |
| *5* | Plum Blossom Needle (Topic) and Preprint Citation Index (Exclude – Database) and Research Commons (Exclude – Database) |
| *6* | RCT (Topic) OR Randomized Controlled Trial (Topic) OR rct (Topic) OR Randomized Controlled Trial (Topic) OR Randomized Controlled Study (Topic) and Preprint Citation Index (Exclude – Database) and Research Commons (Exclude – Database) |
| *7* | #2 OR #3 OR #4 OR #5 and Preprint Citation Index (Exclude – Database) and Research Commons (Exclude – Database) |
| *8* | #1 AND #7 AND #6 and Preprint Citation Index (Exclude – Database) and Research Commons (Exclude – Database) |

Appendix 3:

3.1List of data extracted from the included randomized clinical trials

| Data Typed | List of Variables |
| --- | --- |
| Research | Lead author, Year of publication, Study duration, Total number of patients in each group |
| Patient | Sex, Age, Duration of disease |
| Intervention | Type of intervention, Timing of main intervention |
| Outcome Indicator | Overall Effectiveness Rate, VAS Visual Analog Scale, Apparent Healing Rate, CMS Score |
| Adverse event | Adverse event |

| **Diagnostic Key Points** | **Description** |
| --- | --- |
| Medical History | The disease usually has a chronic onset, often triggered by trauma, exposure to cold, or other external factors. It predominantly affects individuals over 40 years of age, with a higher prevalence in females. |
| Clinical Manifestations | (1) Pain: Shoulder pain is more pronounced at night; (2) Tenderness: Diffuse tenderness around the shoulder joint, sometimes involving the trapezius or interscapular region; (3) Movement Limitation: Restriction of motion in all directions, most notably in abduction and external rotation; (4) Muscle Atrophy: Some patients may exhibit disuse atrophy of the supraspinatus and deltoid muscles. |
| Imaging Findings | In the acute phase, X-rays often appear normal, though some patients may show supraspinatus tendon calcification or local osteoporosis. MRI may reveal abnormal signals in the supraspinatus, infraspinatus, or long head of the biceps tendon, as well as thickening of the coracohumeral ligament and rotator interval, or the subcoracoid “triangle sign.” |

Appendix3 3.2 Diagnostic Criteria for Frozen Shoulder (Chinese Medical Association)

Appendix 4: Risk of bias of randomized clinical trials

Table S4: Study level risk of bias assessment using Cochrane risk of bias tool 2.0 for assessing risk of bias of randomized clinical trials.

| Study ID | Randomization process | Deviations from intended interventions | Mising outcome data | Measurement of the outcome | Selection of the reported result | Overall Bias |
| --- | --- | --- | --- | --- | --- | --- |
| Yang Bai 2020 | Low | Some concerns | Low | Low | Low | High |
| Xingang Bao 2017 | Low | Some concerns | Low | Low | Low | Low |
| Tao Che 2006 | Low | Some concerns | Low | Low | Low | Low |
| Zhiwu Chen 2010 | Low | Some concerns | Low | Low | Low | Low |
| Huizhen Chen 2006 | Some concerns | Some concerns | Low | Low | Low | Low |
| Libin Chen 2020 | Low | Low | Low | Low | Low | Low |
| Shuo Chen 2016 | Low | Some concerns | Low | Low | Low | Low |
| Yingchun Chen 2015 | Low | Some concerns | Low | Low | Low | Some concerns |
| Yong Cheng 2005 | Low | Some concerns | Low | Some concerns | Some concerns | High |
| Yanxi Deng 2007 | Low | Some concerns | Low | Low | Low | Low |
| Liqun Dong 2014 | Low | Some concerns | Low | Low | Low | Low |
| Chunhai Fan 2015 | Low | Low | Low | Low | Low | Low |
| Jiagui Fan 2005 | Low | Low | Low | Low | Low | Some concerns |
| Zhengen Feng 2016 | Low | Low | Low | Some concerns | Some concerns | Low |
| Chenglin Zhu 2024 | Low | Some concerns | Low | Low | Low | High |
| Jingyu Guan 2010 | Some concerns | Some concerns | Low | Low | Low | Low |
| Changqing Guo 2007 | Low | Some concerns | Low | Low | Low | High |
| Li He 2019 | Some concerns | Some concerns | Low | Low | Low | High |
| Yucai He 2019 | Low | Some concerns | Low | Low | Low | Some concerns |
| Rongjuan Hou 2013 | Low | Some concerns | Low | Low | Low | Low |
| Ping Shao 2006 | Low | Some concerns | Low | Low | Low | High |
| ShuiRong Hu 2008 | Some concerns | Some concerns | Low | Low | Low | Low |
| Lianxin Huang2013 | Low | Some concerns | Low | Low | Low | Low |
| Zhao Huang 2012 | Low | Some concerns | Low | Low | Low | Some concerns |
| Jinguo Ji 2019 | Low | Some concerns | Low | Some concerns | Some concerns | Some concerns |
| DeCong Kong2018 | Low | Some concerns | Some concerns | Some concerns | Some concerns | Low |
| Peizheng Lei 2013 | Low | Some concerns | Some concerns | Low | Low | Some concerns |
| Junhe Li 2019 | Low | Some concerns | Some concerns | Some concerns | Some concerns | Low |
| Xinwei Li 2017 | Low | Some concerns | Some concerns | Low | Low | Low |
| Yan LI 2006 | Low | Some concerns | Low | Low | Low | Low |
| Lingyan Ling2019 | Low | Some concerns | Low | Low | Low | Low |
| Xinxiao Lin 2016 | Low | Some concerns | Low | Low | Low | Low |
| Ziling Lin 2011 | Low | Some concerns | Low | Low | Low | Low |
| Jianwei Ling 2013 | Some concerns | Low | Low | Low | Low | Low |
| Ming Liu 2014 | Low | Some concerns | Low | Low | Low | Low |
| Yan Shao 2017 | Low | Some concerns | Low | Low | Low | Low |
| Yuanyuan Liu 2007 | Some concerns | Some concerns | Low | Low | Low | Low |
| Weiping Luo 2006 | Low | Some concerns | Low | Low | Low | High |
| LiHui Ma 2022 | Low | Some concerns | Low | Low | Low | High |
| Wei Ming 2018 | Low | Some concerns | Low | Low | Low | Some concerns |
| Shangxi Pu 2017 | Low | Some concerns | Low | Some concerns | Some concerns | Low |
| Qiao Ye 2019 | Low | Some concerns | Low | Low | Low | Low |
| Si Chen 2016 | Low | Some concerns | Low | Low | Low | Low |
| Hui Shi 2012 | High | Some concerns | Low | Low | Low | Low |
| BiaoMin Qin 2006 | Low | Some concerns | Low | Low | Low | Low |
| Chenyao Wang 2011 | Some concerns | Some concerns | Low | Low | Low | Low |
| Fengchuan Wang 2008 | Low | Some concerns | Low | Low | Low | Low |
| Hongwei Wang 2006 | Low | Some concerns | Low | Low | Low | Low |
| Hongguo Wang 2022 | Low | Some concerns | Low | Low | Low | Low |
| Hui Wang 2020 | Low | Some concerns | Low | Low | Low | Low |
| Xi Wen 2018 | Low | Low | Low | Low | Low | Low |
| Bin Wu 2018 | Low | Low | Low | Low | Low | Low |
| Chengju Wu 2007 | Low | Low | Low | Low | Low | Low |
| Guowei Wu 2013 | Low | Some concerns | Low | Low | Low | Low |
| Xianzhao Lin 2023 | Low | Some concerns | Low | Low | Low | Low |
| Xiangwei Xu 2020 | Low | Some concerns | Low | Some concerns | Some concerns | Low |
| Kaisheng Xu 2009 | Low | Some concerns | Low | Low | Low | Low |
| Hongwei Yan 2016 | Low | Some concerns | Low | Low | Some concerns | Low |
| Xuejun Yang 2014 | Low | Some concerns | Low | Low | Low | Low |
| Guanghao Ma 2006 | Low | Low | Low | Low | Low | Low |
| Qiong Zhang 1933, | Low | Some concerns | Some concerns | Low | Low | Low |
| Ruilian Zhang 2012 | Low | Some concerns | Low | Low | Some concerns | Low |
| Minming Zhao 2017 | Low | Low | Low | Low | Low | High |
| TianYi Zhou 2023 | Low | Low | Low | Low | Low | Some concerns |
| Xiaoping Zhou 2007 | Low | Some concerns | Low | Low | Some concerns | Low |
| Wang, H. 2025 | Low | Low | Low | Low | Low | Low |
| Zhou, T. Y. 2023 | Low | Low | Low | High | Low | Some concerns |
| Lu, J. 2008 | Some concerns | Low | Low | High | Low | Some concerns |
| Wang, B. B. 2020 | Low | Some concerns | Low | Low | Low | Some concerns |
| Chen, M. Y. 2013 | Low | Some concerns | Low | Some concerns | Low | Some concerns |

Appendix 5: Evaluation of inconsistency and heterogeneity

Table S5.1: consistency

| Outcome Indicator | Chi square | P value | τ^2^ |
| --- | --- | --- | --- |
| Overall Effectiveness Rate | 29.24 | 0.1083 | 0.000231 |
| VAS | 16.38 | 0.3574 | 0.242314 |
| Apparent Healing Rate | 22.46 | 0.0696 | 0.002123 |
| CMS | 4.65 | 0.3111 | 0.341250 |
| Adverse Reactions and Shedding | 1.34 | 0.2479 | 0.000014 |

Table S5.2: Side-splitting of total effective rate：Inconsistency test between direct and indirect

treatment comparisons in mixed treatment comparison.

| Side |  |  | Direct |  | Indirect |  |  | Difference | tau |
| --- | --- | --- | --- | --- | --- | --- | --- | --- | --- |
|  |  |  | Coef. | Std.Err. | Coef. | Std.Err. | Coef. | Std.Err. | P>\|z\| |
|  | A | B | 0.170059 | 0.0935026 | -0.001308 | 0.0546207 | 0.171367 | 0.1082873 | 0.114 |
|  | A | C | 0.0568381 | 0.072868 | 0.0276877 | 0.0613791 | 0.0291504 | 0.0952666 | 0.76 |
|  | A | D | 0.1451853 | 0.0746564 | 0.0935815 | 0.0520183 | 0.0516038 | 0.0908593 | 0.57 |
|  | A | E | 0.118937 | 0.0632667 | 0.3249313 | 0.0701537 | -0.2059943 | 0.094434 | 0.029 |
|  | A | F | . | . | . | . | . | . | . |
|  | A | G | 0.1032691 | 0.1179219 | -0.0430121 | 0.0907473 | 0.1462813 | 0.1487973 | 0.326 |
|  | A | H | 0.3339257 | 0.1236866 | 0.0515922 | 0.1043171 | 0.2823334 | 0.1618974 | 0.081 |
|  | A | I | . | . | . | . | . | . | . |
|  | A | K | 0.0327898 | 0.1061055 | 0.2912292 | 0.0881939 | -0.2584394 | 0.137973 | 0.061 |
|  | A | M | -0.0779076 | 0.0915926 | -0.1343879 | 0.0615878 | 0.0564803 | 0.1101958 | 0.608 |
|  | A | N | 0.1133287 | 0.1424897 | -0.0100689 | 0.0796461 | 0.1233975 | 0.1632385 | 0.45 |
|  | A | O | -0.3053816 | 0.1517233 | -0.0885248 | 0.0603679 | -0.2168569 | 0.1632919 | 0.184 |
|  | A | P | -0.1978257 | 0.1485575 | 0.1263546 | 0.1138339 | -0.3241803 | 0.1871563 | 0.083 |
|  | B | C | 0.1032697 | 0.0962298 | -0.0485643 | 0.0633268 | 0.1518341 | 0.1151988 | 0.187 |
|  | B | D | 0.068506 | 0.088158 | 0.0677039 | 0.0622531 | 0.0008021 | 0.1078759 | 0.994 |
|  | B | E | 0.369747 | 0.1481071 | 0.1308251 | 0.0663004 | 0.2389219 | 0.1622697 | 0.141 |
|  | B | H | 0.0327898 | 0.1197833 | 0.2065982 | 0.109545 | -0.1738084 | 0.1623211 | 0.284 |
|  | B | J | 0.0512933 | 0.1277289 | 0.0089022 | 0.1245013 | 0.0423911 | 0.1783683 | 0.812 |
|  | B | M | -0.1654603 | 0.0819363 | -0.1555571 | 0.0669062 | -0.0099033 | 0.1054722 | 0.925 |
|  | C | D | 0.2043374 | 0.1095189 | 0.032894 | 0.057781 | 0.1714434 | 0.1237702 | 0.166 |
|  | C | E | 0.14842 | 0.1390244 | 0.1804754 | 0.068072 | -0.0320554 | 0.1547953 | 0.836 |
|  | C | L | 0.1440644 | 0.0704442 | 0.0647789 | 115.668 | 0.0792856 | 115.668 | 0.999 |
|  | C | M | -0.0711236 | 0.1129117 | -0.1842505 | 0.0645309 | 0.1131269 | 0.1300511 | 0.384 |
|  | C | O | -0.1789569 | 0.1020866 | -0.1470958 | 0.0736298 | -0.0318612 | 0.1258663 | 0.8 |
|  | D | E | 0.3298824 | 0.1157332 | 0.0422717 | 0.059945 | 0.2876107 | 0.1303364 | 0.027 |
|  | D | K | 0.1372011 | 0.1329563 | 0.0499165 | 0.0902289 | 0.0872847 | 0.1606818 | 0.587 |
|  | D | M | -0.3262089 | 0.109891 | -0.1956687 | 0.0610401 | -0.1305403 | 0.1256398 | 0.299 |
|  | D | N | -0.2170644 | 0.1388639 | -0.044209 | 0.0844537 | -0.1728554 | 0.1625288 | 0.288 |
|  | D | O | -0.1870553 | 0.0940495 | -0.253019 | 0.0720824 | 0.0659637 | 0.1184467 | 0.578 |
|  | D | P | -8.59E-10 | 0.105407 | -0.3241804 | 0.1546507 | 0.3241804 | 0.1871563 | 0.083 |
|  | E | N | -0.1838435 | 0.0765515 | -0.218229 | 0.1134675 | 0.0343855 | 0.1368383 | 0.802 |
|  | G | K | 0.2237845 | 0.0711096 | 0.0295679 | 0.1235838 | 0.1942166 | 0.1425218 | 0.173 |
|  | G | O | -0.1790482 | 0.1375158 | -0.1036252 | 0.0998758 | -0.075423 | 0.1699582 | 0.657 |
|  | H | O | -0.1823215 | 0.1601695 | -0.3340584 | 0.1054394 | 0.151737 | 0.1917596 | 0.429 |
|  | J | M | -0.1723193 | 0.1118457 | -0.2147101 | 0.138903 | 0.0423909 | 0.1783683 | 0.812 |
|  | M | O | 0.04652 | 0.113296 | -0.0220098 | 0.0745377 | 0.0685298 | 0.1356166 | 0.613 |

Table S5.3: Side-splitting of VAS. Inconsistency test between direct and indirect treatment comparisons in mixed treatment comparison

| Side |  | Direct |  | Indirect |  | Difference |  | tau |
| --- | --- | --- | --- | --- | --- | --- | --- | --- |
|  |  | Coef. | Std.Err. | Coef. | Std.Err. | Coef. | Std.Err. | P>\|z\| |
| A | B | -1.09874 | 0.7324801 | 0.6155554 | 0.5975194 | -1.714296 | 0.9452753 | 0.07 |
| A | D | -1.068997 | 0.785434 | -1.535845 | 0.6904623 | 0.4668477 | 1.045776 | 0.655 |
| A | E | -1.56 | 1.118964 | -2.059324 | 0.7616898 | 0.4993241 | 1.353607 | 0.712 |
| A | F | . | . | . | . | . | . | . |
| A | I | -1.404338 | 0.7996913 | -1.339924 | 0.9664985 | -0.0644142 | 1.254421 | 0.959 |
| A | J | . | . | . | . | . | . | . |
| A | N | 1.99 | 1.079199 | 0.1912594 | 0.6767642 | 1.79874 | 1.273845 | 0.158 |
| B | C | -0.856047 | 0.7852628 | -0.2054115 | 0.705073 | -0.6506355 | 1.055355 | 0.538 |
| B | D | -1.958205 | 0.7657338 | -0.8163767 | 0.6158429 | -1.141829 | 0.9826831 | 0.245 |
| B | E | -2.539998 | 1.124025 | -1.550668 | 0.7127156 | -0.9893296 | 1.330937 | 0.457 |
| B | H | -0.46 | 1.081639 | -0.3247076 | 199.978 | -0.1352924 | 199.9807 | 0.999 |
| B | I | 0.0299998 | 1.029855 | -2.060855 | 0.7732029 | 2.090855 | 1.287806 | 0.104 |
| B | K | -0.8599983 | 1.102658 | 0.4557667 | 1.243626 | -1.315765 | 1.662064 | 0.429 |
| B | N | 0.6099998 | 1.168119 | 0.8103477 | 0.6208848 | -0.2003478 | 1.322875 | 0.88 |
| C | D | -0.5300021 | 1.113748 | -0.8565738 | 0.6731747 | 0.3265717 | 1.301383 | 0.802 |
| C | E | -0.7400137 | 1.214561 | -1.582368 | 0.7752071 | 0.8423546 | 1.44087 | 0.559 |
| C | M | -0.8899994 | 1.051524 | 1.372886 | 1.221208 | -2.262885 | 1.611537 | 0.16 |
| C | N | 1.039998 | 1.120054 | 1.342623 | 0.6729797 | -0.3026253 | 1.306684 | 0.817 |
| C | O | 0.7899977 | 1.136736 | 1.172895 | 0.8812967 | -0.3828969 | 1.43835 | 0.79 |
| D | E | -2.080001 | 1.035148 | 0.0971626 | 0.6888998 | -2.177163 | 1.243428 | 0.08 |
| D | L | -0.6400061 | 1.099839 | -2.00521 | 1.244757 | 1.365204 | 1.661042 | 0.411 |
| D | N | 2.349998 | 1.093867 | 1.905638 | 0.6878187 | 0.4443598 | 1.292145 | 0.731 |
| E | G | -1.700001 | 1.015696 | 1.065471 | 1.050105 | -2.765471 | 1.460944 | 0.058 |
| G | L | -0.6138199 | 0.7831407 | 0.7522952 | 1.465062 | -1.366115 | 1.661107 | 0.411 |
| G | O | 1.869984 | 1.128676 | 3.620856 | 1.149997 | -1.750872 | 1.611337 | 0.277 |
| I | O | 3.169996 | 1.032307 | 0.6673134 | 0.9724873 | 2.502683 | 1.418235 | 0.078 |
| K | N | 0.4699988 | 1.101034 | 1.785807 | 1.245082 | -1.315808 | 1.66208 | 0.429 |
| M | N | 0.1999986 | 1.064885 | 2.462875 | 1.209576 | -2.262876 | 1.611539 | 0.16 |
| N | O | -0.5699965 | 1.116297 | -0.0181631 | 0.891758 | -0.5518334 | 1.428758 | 0.699 |

Table S5.4: Side-splitting of apparent healing rate. Inconsistency test between direct and indirect treatment comparisons in mixed treatment comparison.

| Side |  | Direct |  | Indirect |  | Difference |  | tau |
| --- | --- | --- | --- | --- | --- | --- | --- | --- |
|  |  | Coef. | Std.Err. | Coef. | Std.Err. | Coef. | Std.Err. | P>\|z\| |
| A | B | 1.13498 | 0.3658087 | 0.1633522 | 0.1485247 | 0.9716277 | 0.3948109 | 0.014 |
| A | C | 0.1268674 | 0.2128757 | 0.5585443 | 0.1998501 | -0.4316769 | 0.2906286 | 0.137 |
| A | D | 0.6771254 | 0.2071795 | 0.3135862 | 0.1476546 | 0.3635392 | 0.2541228 | 0.153 |
| A | E | 0.5153708 | 0.2313293 | 0.0310061 | 0.4484756 | 0.4843647 | 0.504588 | 0.337 |
| A | F | . | . | . | . | . | . | . |
| A | H | 0.4789947 | 0.2421774 | 0.3638494 | 0.2992009 | 0.1151453 | 0.3837564 | 0.764 |
| A | J | 0.112478 | 0.2792898 | 0.888889 | 0.2684893 | -0.7764111 | 0.3874136 | 0.045 |
| A | K | -0.2495591 | 0.2190773 | -0.2736622 | 0.2326466 | 0.0241031 | 0.3203722 | 0.94 |
| A | M | -0.4895482 | 0.3251294 | 0.0475331 | 0.1833961 | -0.5370813 | 0.373287 | 0.15 |
| A | N | -0.1978257 | 0.2974814 | -0.0705033 | 0.3656455 | -0.1273224 | 0.4713723 | 0.787 |
| B | C | 0.3096141 | 0.2239832 | -0.1964271 | 0.2163094 | 0.5060412 | 0.3113795 | 0.104 |
| B | D | 0.2887075 | 0.2245566 | 0.0055169 | 0.1925838 | 0.2831906 | 0.2959154 | 0.339 |
| B | H | 1.19E-06 | 0.3719162 | 0.1733857 | 0.2541879 | -0.1733845 | 0.4504809 | 0.7 |
| B | I | 0.0741084 | 0.3230965 | -0.0209617 | 0.3152284 | 0.0950701 | 0.451398 | 0.833 |
| B | K | -0.7268705 | 0.2607488 | -0.4797268 | 0.2068582 | -0.2471437 | 0.3321143 | 0.457 |
| C | D | 5.98E-07 | 0.3263128 | 0.1018985 | 0.1843803 | -0.1018979 | 0.3748015 | 0.786 |
| C | M | -0.3254216 | 0.3568896 | -0.4897674 | 0.2235671 | 0.1643457 | 0.4211323 | 0.696 |
| D | J | 0.3227738 | 0.3275798 | -0.0689337 | 0.2632844 | 0.3917076 | 0.4202703 | 0.351 |
| D | K | -0.5306276 | 0.3403696 | -0.7587304 | 0.1979326 | 0.2281029 | 0.3937369 | 0.562 |
| D | L | -0.6131021 | 0.3802639 | -0.1287532 | 0.3316847 | -0.4843489 | 0.504594 | 0.337 |
| D | M | -0.2502774 | 0.2083225 | -0.7857182 | 0.2107163 | 0.5354408 | 0.2966087 | 0.071 |
| D | N | -0.5212962 | 0.3413148 | -0.6486232 | 0.3251095 | 0.1273271 | 0.4713723 | 0.787 |
| E | L | -0.2364487 | 0.2005272 | -0.7208151 | 0.4630368 | 0.4843664 | 0.5045943 | 0.337 |
| G | J | 0.4411082 | 0.2034825 | -0.1272876 | 0.4237045 | 0.5683959 | 0.4701016 | 0.227 |
| G | M | -0.5500459 | 0.3305523 | 0.018357 | 0.3342609 | -0.5684028 | 0.4701014 | 0.227 |
| H | M | -0.5240692 | 0.3870476 | -0.5090942 | 0.2646273 | -0.0149751 | 0.468864 | 0.975 |
| I | K | -0.5705653 | 0.2595799 | -0.6656258 | 0.3695829 | 0.0950605 | 0.4513976 | 0.833 |

Table S5.5: Side-splitting of CMS. Inconsistency test between direct and indirect treatment comparisons in mixed treatment comparison.

| Side |  | Direct |  | Indirect |  | Difference |  | tau |
| --- | --- | --- | --- | --- | --- | --- | --- | --- |
|  |  | Coef. | Std.Err. | Coef. | Std.Err. | Coef. | Std.Err. | P>\|z\| |
| A | B | 1.077657 | 0.277538 | -0.4005486 | 0.6267524 | 1.478206 | 0.6854188 | 0.031 |
| A | C | -0.8361562 | 0.179045 | 0.6419488 | 0.6626586 | -1.478105 | 0.686325 | 0.031 |
| B | D | 2.010874 | 0.3108421 | 0.532414 | 0.6106745 | 1.47846 | 0.6851804 | 0.031 |
| C | F | -1.883269 | 0.2000944 | -0.4068515 | 0.6582753 | -1.476418 | 0.6875477 | 0.032 |
| D | G | -2.804053 | 0.4331759 | -4.281869 | 0.5306613 | 1.477816 | 0.6849999 | 0.031 |
| E | F | -4.391703 | 0.6779164 | -0.1758745 | 200.0275 | -4.215829 | 200.0295 | 0.983 |
| F | G | 1.526075 | 0.1893374 | 3.003702 | 0.658713 | -1.477627 | 0.6854957 | 0.031 |

Table S5.6: Side-splitting of adverse reactions. Inconsistency test between direct and indirect treatment comparisons in mixed treatment comparison

| Side |  | Direct |  | Indirect |  | Difference |  | tau |
| --- | --- | --- | --- | --- | --- | --- | --- | --- |
|  |  | Coef. | Std.Err. | Coef. | Std.Err. | Coef. | Std.Err. | P>\|z\| |
| A | E | . | . | . | . | . | . | . |
| A | G | 1.703257 | 1.665251 | 1.381162 | 40.83037 | 0.3220948 | 40.86432 | 0.994 |
| B | C | -0.7490561 | 1.111368 | -0.844794 | 81.69288 | 0.0957378 | 81.69601 | 0.999 |
| B | D | 0.6931472 | 1.356923 | -0.1996812 | 200.0386 | 0.8928283 | 200.0355 | 0.996 |
| B | F | -0.6509862 | 0.7638245 | -1.543815 | 199.9527 | 0.8928283 | 199.9528 | 0.996 |
| B | G | 1.256361 | 0.7387198 | 1.581838 | 43.6854 | -0.3254769 | 43.6919 | 0.994 |
| C | H | 1.466337 | 1.269426 | 2.072588 | 200.0207 | -0.6062507 | 200.0182 | 0.998 |

Appendix6: CINeMA Assessment

We use the CINeMA framework to evidence certainty, assessing it for each network estimate based on the following criteria:

- Within study bias: We classified the overall risk of bias for each study as low risk of bias, the risk of bias as moderate when none of the four assessed risk of bias items were rated as high risk, and the risk of bias as high when one or both items were rated as high risk. See Appendix 4 for the bias assessment. The risk of bias for a pairwise comparison of each drug is shown in figure S8.1-8.2.
- Reporting bias: We judged it visually by a funnel plot (Appendix 9).
- Indirectness: Transferability assumptions were assessed by reporting the mean total prior miscarriages in the included study population and by comparing age at baseline concordance between groups.
- Imprecision: We use the CINeMA website to grade the accuracy of each comparison.
- Heterogeneity: We assessed the degree of worry by comparing clinical reasoning based on 95% confidence intervals (CIs) while applying the same clinical reasoning framework as for inaccuracy. In particular, we judged the consistency of our findings based on the confidence and prediction intervals associated with clinically important effect sizes. And we used the same thresholds of clinical significance as described above and followed the recommendations automatically provided by CINeMA (https://cinema.ispm.unibe.ch/).

Figure S6.1: Overall risk of bias by treatment comparison in total effective rate.


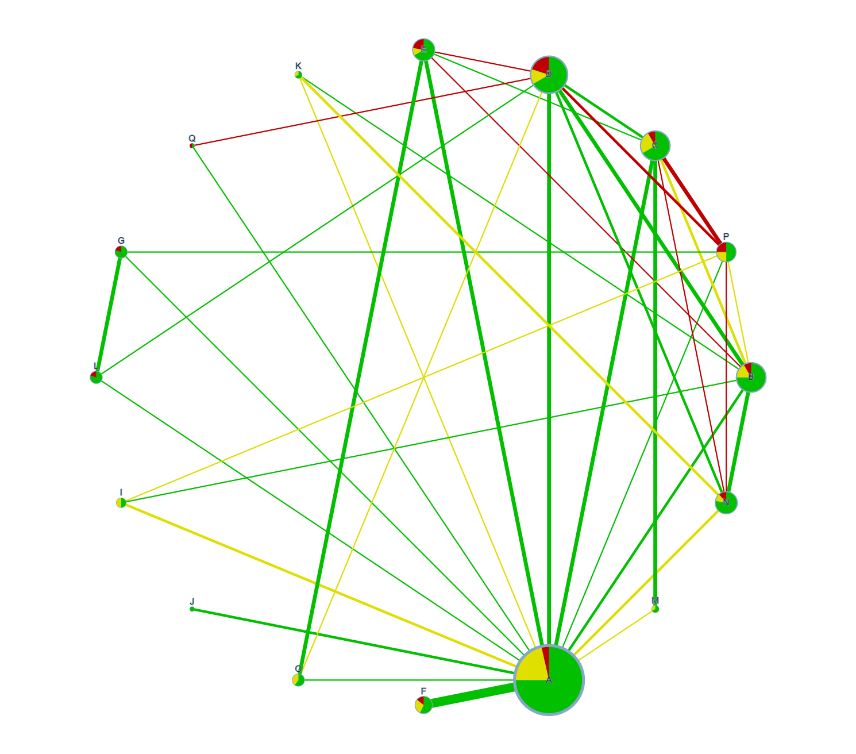


Table S6.2: Overall risk of bias by treatment comparison in total effective rate


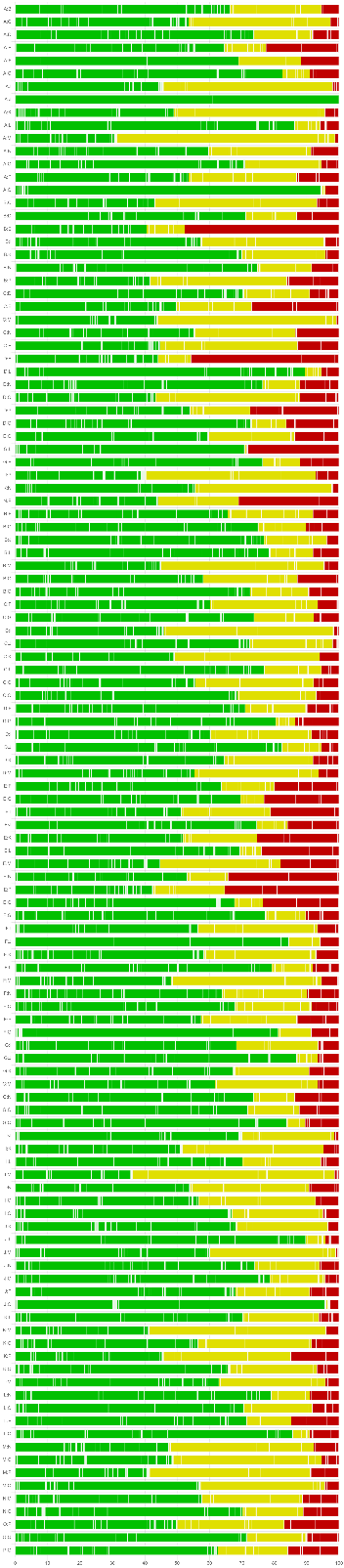


Table S6.3: CINeMA Results of total effective rate

| Comparison | Within-study bias | Reporting bias | Indirectness | Imprecision | Heterogeneity | Incoherence | Confidence rating |
| --- | --- | --- | --- | --- | --- | --- | --- |
| A:B | No concerns | Low risk | No concerns | Major concerns | No concerns | Major concerns | Low |
| A:C | No concerns | Low risk | No concerns | No concerns | Major concerns | No concerns | Low |
| A:D | No concerns | Low risk | No concerns | No concerns | No concerns | No concerns | High |
| A:E | No concerns | Low risk | No concerns | No concerns | No concerns | No concerns | High |
| A:F | No concerns | Low risk | No concerns | No concerns | No concerns | Major concerns | Low |
| A:G | No concerns | Low risk | No concerns | No concerns | No concerns | No concerns | High |
| A:I | Some concerns | Low risk | No concerns | No concerns | No concerns | No concerns | Moderate |
| A:J | No concerns | Low risk | No concerns | Major concerns | No concerns | Major concerns | Low |
| A:K | No concerns | Low risk | No concerns | Major concerns | No concerns | No concerns | Low |
| A:L | No concerns | Low risk | No concerns | No concerns | No concerns | No concerns | High |
| A:M | Some concerns | Low risk | No concerns | No concerns | No concerns | No concerns | Moderate |
| A:N | No concerns | Low risk | No concerns | No concerns | Major concerns | No concerns | Low |
| A:O | No concerns | Low risk | No concerns | Major concerns | No concerns | No concerns | Low |
| A:P | No concerns | Low risk | No concerns | Major concerns | No concerns | Major concerns | Low |
| A:Q | No concerns | Low risk | No concerns | Major concerns | No concerns | No concerns | Low |
| B:C | Some concerns | Low risk | No concerns | Major concerns | No concerns | No concerns | Low |
| B:D | No concerns | Low risk | No concerns | No concerns | Major concerns | No concerns | Low |
| B:E | Major concerns | Low risk | No concerns | No concerns | No concerns | No concerns | Low |
| B:I | No concerns | Low risk | No concerns | No concerns | No concerns | No concerns | High |
| B:K | No concerns | Low risk | No concerns | Major concerns | No concerns | No concerns | Low |
| B:N | No concerns | Low risk | No concerns | No concerns | No concerns | No concerns | High |
| B:P | Some concerns | Low risk | No concerns | No concerns | Major concerns | No concerns | Low |
| C:D | No concerns | Low risk | No concerns | Major concerns | No concerns | No concerns | Low |
| C:E | No concerns | Low risk | No concerns | No concerns | No concerns | No concerns | High |
| C:M | Some concerns | Low risk | No concerns | No concerns | No concerns | Major concerns | Low |
| C:N | No concerns | Low risk | No concerns | No concerns | No concerns | No concerns | High |
| C:P | No concerns | Low risk | No concerns | No concerns | No concerns | No concerns | High |
| D:E | Major concerns | Low risk | No concerns | No concerns | No concerns | No concerns | Low |
| D:L | No concerns | Low risk | No concerns | Major concerns | No concerns | No concerns | Low |
| D:N | No concerns | Low risk | No concerns | No concerns | No concerns | No concerns | High |
| D:O | Some concerns | Low risk | No concerns | Major concerns | No concerns | No concerns | Low |
| D:P | No concerns | Low risk | No concerns | No concerns | No concerns | No concerns | High |
| D:Q | No concerns | Low risk | No concerns | No concerns | No concerns | No concerns | High |
| E:O | No concerns | Low risk | No concerns | No concerns | No concerns | No concerns | High |
| G:L | No concerns | Low risk | No concerns | Major concerns | No concerns | No concerns | Low |
| G:P | No concerns | Low risk | No concerns | No concerns | No concerns | No concerns | High |
| I:P | Some concerns | Low risk | No concerns | No concerns | No concerns | No concerns | Moderate |
| K:N | No concerns | Low risk | No concerns | No concerns | Major concerns | No concerns | Low |
| N:P | No concerns | Low risk | No concerns | Major concerns | No concerns | No concerns | Low |
| B:F | No concerns | Low risk | No concerns | Major concerns | No concerns | Major concerns | Low |
| B:G | No concerns | Low risk | No concerns | No concerns | Major concerns | Major concerns | Low |
| B:J | No concerns | Low risk | No concerns | Major concerns | No concerns | Major concerns | Low |
| B:L | No concerns | Low risk | No concerns | No concerns | No concerns | Major concerns | Low |
| B:M | Some concerns | Low risk | No concerns | No concerns | No concerns | Major concerns | Low |
| B:O | No concerns | Low risk | No concerns | Major concerns | No concerns | Major concerns | Low |
| B:Q | No concerns | Low risk | No concerns | Major concerns | No concerns | Major concerns | Low |
| C:F | No concerns | Low risk | No concerns | Major concerns | No concerns | Major concerns | Low |
| C:G | No concerns | Low risk | No concerns | Major concerns | No concerns | Major concerns | Low |
| C:I | Some concerns | Low risk | No concerns | No concerns | Major concerns | Major concerns | Low |
| C:J | No concerns | Low risk | No concerns | Major concerns | No concerns | Major concerns | Low |
| C:K | No concerns | Low risk | No concerns | Major concerns | No concerns | Major concerns | Low |
| C:L | No concerns | Low risk | No concerns | No concerns | No concerns | Major concerns | Low |
| C:O | No concerns | Low risk | No concerns | Major concerns | No concerns | Major concerns | Low |
| C:Q | No concerns | Low risk | No concerns | No concerns | Major concerns | Major concerns | Low |
| D:F | No concerns | Low risk | No concerns | Major concerns | No concerns | Major concerns | Low |
| D:G | No concerns | Low risk | No concerns | Major concerns | No concerns | Major concerns | Low |
| D:I | No concerns | Low risk | No concerns | Major concerns | No concerns | Major concerns | Low |
| D:J | No concerns | Low risk | No concerns | Major concerns | No concerns | Major concerns | Low |
| D:K | No concerns | Low risk | No concerns | No concerns | Major concerns | Major concerns | Low |
| D:M | No concerns | Low risk | No concerns | No concerns | Major concerns | Major concerns | Low |
| E:F | No concerns | Low risk | No concerns | No concerns | No concerns | Major concerns | Low |
| E:G | No concerns | Low risk | No concerns | Major concerns | No concerns | Major concerns | Low |
| E:I | No concerns | Low risk | No concerns | Major concerns | No concerns | Major concerns | Low |
| E:J | No concerns | Low risk | No concerns | No concerns | No concerns | Major concerns | Low |
| E:K | No concerns | Low risk | No concerns | No concerns | No concerns | Major concerns | Low |
| E:L | No concerns | Low risk | No concerns | Major concerns | No concerns | Major concerns | Low |
| E:M | No concerns | Low risk | No concerns | Major concerns | No concerns | Major concerns | Low |
| E:N | No concerns | Low risk | No concerns | No concerns | No concerns | Major concerns | Low |
| E:P | No concerns | Low risk | No concerns | No concerns | No concerns | Major concerns | Low |
| E:Q | No concerns | Low risk | No concerns | No concerns | No concerns | Major concerns | Low |
| F:G | No concerns | Low risk | No concerns | Major concerns | No concerns | Major concerns | Low |
| F:I | No concerns | Low risk | No concerns | Major concerns | No concerns | Major concerns | Low |
| F:J | No concerns | Low risk | No concerns | Major concerns | No concerns | Major concerns | Low |
| F:K | No concerns | Low risk | No concerns | Major concerns | No concerns | Major concerns | Low |
| F:L | No concerns | Low risk | No concerns | Major concerns | No concerns | Major concerns | Low |
| F:M | No concerns | Low risk | No concerns | Major concerns | No concerns | Major concerns | Low |
| F:N | No concerns | Low risk | No concerns | No concerns | No concerns | Major concerns | Low |
| F:O | No concerns | Low risk | No concerns | Major concerns | No concerns | Major concerns | Low |
| F:P | No concerns | Low risk | No concerns | No concerns | No concerns | Major concerns | Low |
| F:Q | No concerns | Low risk | No concerns | No concerns | No concerns | Major concerns | Low |
| G:I | No concerns | Low risk | No concerns | Major concerns | No concerns | Major concerns | Low |
| G:J | No concerns | Low risk | No concerns | Major concerns | No concerns | Major concerns | Low |
| G:K | No concerns | Low risk | No concerns | No concerns | No concerns | Major concerns | Low |
| G:M | No concerns | Low risk | No concerns | Major concerns | No concerns | Major concerns | Low |
| G:N | No concerns | Low risk | No concerns | No concerns | No concerns | Major concerns | Low |
| G:O | No concerns | Low risk | No concerns | No concerns | Major concerns | Major concerns | Low |
| G:Q | No concerns | Low risk | No concerns | No concerns | No concerns | Major concerns | Low |
| I:J | No concerns | Low risk | No concerns | Major concerns | No concerns | Major concerns | Low |
| I:K | No concerns | Low risk | No concerns | No concerns | No concerns | Major concerns | Low |
| I:L | No concerns | Low risk | No concerns | Major concerns | No concerns | Major concerns | Low |
| I:M | Some concerns | Low risk | No concerns | Major concerns | No concerns | Major concerns | Low |
| I:N | No concerns | Low risk | No concerns | No concerns | No concerns | Major concerns | Low |
| I:O | No concerns | Low risk | No concerns | No concerns | Major concerns | Major concerns | Low |
| I:Q | No concerns | Low risk | No concerns | No concerns | No concerns | Major concerns | Low |
| J:K | No concerns | Low risk | No concerns | Major concerns | No concerns | Major concerns | Low |
| J:L | No concerns | Low risk | No concerns | Major concerns | No concerns | Major concerns | Low |
| J:M | No concerns | Low risk | No concerns | Major concerns | No concerns | Major concerns | Low |
| J:N | No concerns | Low risk | No concerns | No concerns | No concerns | Major concerns | Low |
| J:O | No concerns | Low risk | No concerns | Major concerns | No concerns | Major concerns | Low |
| J:P | No concerns | Low risk | No concerns | No concerns | No concerns | Major concerns | Low |
| J:Q | No concerns | Low risk | No concerns | No concerns | No concerns | Major concerns | Low |
| K:L | No concerns | Low risk | No concerns | No concerns | No concerns | Major concerns | Low |
| K:M | Some concerns | Low risk | No concerns | No concerns | No concerns | Major concerns | Low |
| K:O | No concerns | Low risk | No concerns | Major concerns | No concerns | Major concerns | Low |
| K:P | No concerns | Low risk | No concerns | Major concerns | No concerns | Major concerns | Low |
| K:Q | No concerns | Low risk | No concerns | Major concerns | No concerns | Major concerns | Low |
| L:M | No concerns | Low risk | No concerns | Major concerns | No concerns | Major concerns | Low |
| L:N | No concerns | Low risk | No concerns | No concerns | No concerns | Major concerns | Low |
| L:O | No concerns | Low risk | No concerns | No concerns | No concerns | Major concerns | Low |
| L:P | No concerns | Low risk | No concerns | No concerns | No concerns | Major concerns | Low |
| L:Q | No concerns | Low risk | No concerns | No concerns | No concerns | Major concerns | Low |
| M:N | No concerns | Low risk | No concerns | No concerns | No concerns | Major concerns | Low |
| M:O | No concerns | Low risk | No concerns | No concerns | No concerns | Major concerns | Low |
| M:P | Some concerns | Low risk | No concerns | No concerns | No concerns | Major concerns | Low |
| M:Q | No concerns | Low risk | No concerns | No concerns | No concerns | Major concerns | Low |
| N:O | No concerns | Low risk | No concerns | No concerns | Major concerns | Major concerns | Low |
| N:Q | No concerns | Low risk | No concerns | Major concerns | No concerns | Major concerns | Low |
| O:P | No concerns | Low risk | No concerns | Major concerns | No concerns | Major concerns | Low |
| O:Q | No concerns | Low risk | No concerns | Major concerns | No concerns | Major concerns | Low |
| P:Q | No concerns | Low risk | No concerns | Major concerns | No concerns | Major concerns | Low |

Appendix 7: Funnel plots

Figure S7.1: Funnel plot of total effective rate.

Figure S7.2: Funnel plot of VAS

Figure S7.3: Funnel plot of apparent healing rate

Figure S7.4: Funnel plot of CMS

Figure S7.5: Funnel plot of Adverse event

Appendix 8: Network maps

Figure S8.1: Network map of the overall effective rate.

Figure S8.2: Network map of the effect on VAS.

Figure S8.3: Network map of the effect on apparent healing rate.

Figure S8.4: Network map of the effect on adverse reactions.

Figure S8.5: Network map of the effect on CMS.

Appendix 9： Forest plot of the outcomes

Figure S9.1: Forest plot of the effect on overall effective rate rate.

Figure S9.2: Forest plot of the effect on VAS

Figure S9.3: Forest plot of the effect on apparent healing rate

Figure S9.4: Forest plot of the effect on adverse reactions

Figure S9.5: Forest plot of the effect on CMS

Appendix 10: SUCRA and cumulative probability plots

Figure S10.1: Cumulative ranking curve plots of total effective rate in range network.：Higher surface under the curve reflects Higher probability of association with total effective rate。

| Treatment | SUCRA | PrBest | MeanRank |
| --- | --- | --- | --- |
| A | 23.7 | 0 | 12.4 |
| B | 40.8 | 0 | 9.9 |
| C | 44 | 0 | 9.4 |
| D | 66.3 | 0 | 6.1 |
| E | 93.6 | 34 | 2 |
| F | 61.5 | 0 | 6.8 |
| G | 63.3 | 0 | 6.5 |
| I | 74.7 | 0.9 | 4.8 |
| J | 57.2 | 0.3 | 7.4 |
| K | 34.8 | 0 | 10.8 |
| L | 90.4 | 27.8 | 2.4 |
| M | 91.7 | 36.8 | 2.2 |
| N | 5.4 | 0 | 15.2 |
| O | 33.4 | 0 | 11 |
| P | 9.1 | 0 | 14.6 |
| Q | 10.1 | 0 | 14.5 |

Figure S10.2: Cumulative ranking curve plots of VAS in range network.：

Higher surface under the curve reflects Higher probability of association with VAS。

| Treatment | SUCRA | PrBest | MeanRank |
| --- | --- | --- | --- |
| A | 74.2 | 2.8 | 4.6 |
| B | 72.5 | 1.4 | 4.8 |
| C | 55.7 | 0.1 | 7.2 |
| D | 32.9 | 0 | 10.4 |
| E | 18.7 | 0 | 12.4 |
| F | 24.8 | 0.1 | 11.5 |
| G | 13 | 0 | 13.2 |
| H | 56.6 | 11.1 | 7.1 |
| I | 31.8 | 0 | 10.5 |
| J | 64.2 | 6.6 | 6 |
| K | 62 | 5.8 | 6.3 |
| L | 7.9 | 0 | 13.9 |
| M | 57.7 | 3.9 | 6.9 |
| N | 92.4 | 40.2 | 2.1 |
| P | 85.7 | 27.9 | 3 |

Figure S10.3: Cumulative ranking curve plots of apparent healing rate in range network：

Higher urface under the curve reflects Higher probability of association with apparent healing rate.

| Treatment | SUCRA | PrBest | MeanRank |
| --- | --- | --- | --- |
| A | 23.6 | 0 | 10.9 |
| B | 58.3 | 1.1 | 6.4 |
| C | 65.1 | 4.4 | 5.5 |
| D | 76.2 | 6.5 | 4.1 |
| E | 71.7 | 17.9 | 4.7 |
| F | 67.8 | 7.5 | 5.2 |
| G | 45 | 1.1 | 8.1 |
| I | 73.3 | 15.9 | 4.5 |
| K | 62.2 | 10.7 | 5.9 |
| L | 83.3 | 34.2 | 3.2 |
| N | 5.6 | 0 | 13.3 |
| O | 35.7 | 0.7 | 9.4 |
| P | 17.5 | 0 | 11.7 |
| Q | 14.5 | 0 | 12.1 |

Figure S10.4: Cumulative ranking curve plots of Adverse reactions and shedding in range network:

Higher surface under the curve reflects Higher probability of association with Adverse reactions and shedding.

| Treatment | SUCRA | PrBest | MeanRank |
| --- | --- | --- | --- |
| A | 44 | 7.2 | 4.9 |
| B | 51.7 | 0.3 | 4.4 |
| C | 31.4 | 0.5 | 5.8 |
| E | 65.6 | 23.4 | 3.4 |
| F | 24.2 | 3.9 | 6.3 |
| H | 32.7 | 0.8 | 5.7 |
| N | 83.5 | 33.8 | 2.2 |
| P | 66.8 | 30.2 | 3.3 |

Figure S10.5: Cumulative ranking curve plots of CMS in range network:

Higher surface under the curve reflects Higher probability of association with CMS.

| Treatm~t | SUCRA | PrBest | MeanRank |
| --- | --- | --- | --- |
| A | 45.4 | 0 | 4.3 |
| B | 66.5 | 0 | 3 |
| C | 26.4 | 0 | 5.4 |
| I | 94.8 | 68.6 | 1.3 |
| K | 86.5 | 31.4 | 1.8 |
| N | 0 | 0 | 7 |
| P | 30.4 | 0 | 5.2 |

Appendix 11 League Table

Appendix 11.1: League table of Summary Estimates for the total effectiveness of acupuncture and related therapies on scapulohumeral periarthritis from Network Meta-analysis of 65 Trials.

| A |  |  |  |  |  |  |  |  |  |  |  |  |  |  |
| --- | --- | --- | --- | --- | --- | --- | --- | --- | --- | --- | --- | --- | --- | --- |
| 0.58 (0.30,1.10) | B |  |  |  |  |  |  |  |  |  |  |  |  |  |
| 0.53 (0.29,0.95) | 0.91 (0.47,1.78) | C |  |  |  |  |  |  |  |  |  |  |  |  |
| 0.26 (0.14,0.47) | 0.45 (0.23,0.88) | 0.49 (0.26,0.92) | D |  |  |  |  |  |  |  |  |  |  |  |
| 0.06 (0.03,0.13) | 0.10 (0.04,0.24) | 0.11 (0.05,0.27) | 0.23 (0.10,0.51) | E |  |  |  |  |  |  |  |  |  |  |
| 0.29 (0.12,0.71) | 0.50 (0.16,1.52) | 0.55 (0.19,1.62) | 1.12 (0.38,3.31) | 4.90 (1.46,16.42) | F |  |  |  |  |  |  |  |  |  |
| 0.26 (0.07,1.05) | 0.46 (0.11,1.98) | 0.50 (0.12,2.11) | 1.02 (0.26,4.08) | 4.48 (0.96,20.89) | 0.92 (0.18,4.76) | G |  |  |  |  |  |  |  |  |
| 0.33 (0.10,1.09) | 0.58 (0.15,2.24) | 0.64 (0.17,2.39) | 1.30 (0.34,4.88) | 5.69 (1.36,23.81) | 1.16 (0.26,5.15) | 1.27 (0.21,7.80) | I |  |  |  |  |  |  |  |
| 0.70 (0.22,2.21) | 1.22 (0.42,3.55) | 1.34 (0.42,4.30) | 2.73 (0.87,8.52) | 11.95 (3.31,43.16) | 2.44 (0.57,10.52) | 2.67 (0.47,15.09) | 2.10 (0.40,10.90) | J |  |  |  |  |  |  |
| 0.07 (0.02,0.27) | 0.13 (0.03,0.51) | 0.14 (0.04,0.54) | 0.28 (0.08,1.03) | 1.25 (0.29,5.38) | 0.26 (0.05,1.24) | 0.28 (0.13,0.62) | 0.22 (0.04,1.27) | 0.10 (0.02,0.55) | K |  |  |  |  |  |
| 0.06 (0.02,0.24) | 0.11 (0.03,0.44) | 0.12 (0.04,0.40) | 0.25 (0.06,0.96) | 1.08 (0.25,4.74) | 0.22 (0.04,1.11) | 0.24 (0.04,1.56) | 0.19 (0.03,1.13) | 0.09 (0.02,0.48) | 0.87 (0.14,5.28) | L |  |  |  |  |
| 2.56 (1.20,5.47) | 4.46 (2.20,9.03) | 4.88 (2.21,10.81) | 9.94 (4.77,20.71) | 43.59 (16.66,114.02) | 8.90 (2.73,28.94) | 9.72 (2.17,43.59) | 7.66 (1.88,31.22) | 3.65 (1.39,9.58) | 34.88 (8.42,144.49) | 40.21 (9.57,169.02) | M |  |  |  |
| 0.75 (0.25,2.27) | 1.30 (0.40,4.24) | 1.42 (0.44,4.61) | 2.90 (0.96,8.74) | 12.71 (4.18,38.67) | 2.59 (0.62,10.86) | 2.84 (0.51,15.79) | 2.23 (0.44,11.31) | 1.06 (0.23,4.84) | 10.17 (1.96,52.79) | 11.73 (2.19,62.70) | 0.29 (0.08,1.02) | N |  |  |
| 2.08 (0.92,4.68) | 3.61 (1.47,8.86) | 3.95 (1.78,8.78) | 8.04 (3.53,18.34) | 35.27 (12.45,99.90) | 7.20 (2.13,24.30) | 7.87 (1.80,34.42) | 6.20 (1.48,26.06) | 2.95 (0.82,10.61) | 28.23 (6.88,115.84) | 32.54 (7.72,137.13) | 0.81 (0.32,2.06) | 2.77 (0.76,10.19) | O |  |
| 2.22 (0.56,8.73) | 3.86 (0.86,17.29) | 4.22 (0.97,18.49) | 8.60 (1.98,37.24) | 37.70 (7.77,182.94) | 7.69 (1.49,39.86) | 8.41 (1.22,58.06) | 6.63 (1.09,40.39) | 3.15 (0.54,18.58) | 30.17 (4.64,196.29) | 34.78 (5.19,233.11) | 0.86 (0.18,4.09) | 2.97 (0.51,17.09) | 1.07 (0.22,5.20) | P |

Appendix 11.2: League table of Summary Estimates for VAS scores of scapulohumeral periarthritis of acupuncture and related therapies from Network Meta-analysis of 65 Trials.

| A |  |  |  |  |  |  |  |  |  |  |  |  |  |  |
| --- | --- | --- | --- | --- | --- | --- | --- | --- | --- | --- | --- | --- | --- | --- |
| 0.06 (-0.89,1.01) | B |  |  |  |  |  |  |  |  |  |  |  |  |  |
| 0.56 (-0.63,1.74) | 0.49 (-0.52,1.51) | C |  |  |  |  |  |  |  |  |  |  |  |  |
| 1.33 (0.33,2.32) | 1.26 (0.32,2.21) | 0.77 (-0.34,1.88) | D |  |  |  |  |  |  |  |  |  |  |  |
| 1.89 (0.68,3.10) | 1.83 (0.66,3.00) | 1.34 (0.07,2.60) | 0.57 (-0.60,1.74) | E |  |  |  |  |  |  |  |  |  |  |
| 1.64 (0.40,2.88) | 1.58 (0.02,3.14) | 1.09 (-0.63,2.81) | 0.32 (-1.27,1.91) | -0.25 (-1.98,1.48) | F |  |  |  |  |  |  |  |  |  |
| 2.24 (0.62,3.86) | 2.18 (0.60,3.77) | 1.69 (0.05,3.32) | 0.92 (-0.61,2.45) | 0.35 (-1.15,1.85) | 0.60 (-1.44,2.64) | G |  |  |  |  |  |  |  |  |
| 0.52 (-1.80,2.84) | 0.46 (-1.66,2.58) | -0.03 (-2.38,2.31) | -0.80 (-3.12,1.52) | -1.37 (-3.79,1.05) | -1.12 (-3.75,1.51) | -1.72 (-4.37,0.92) | H |  |  |  |  |  |  |  |
| 1.37 (0.19,2.55) | 1.31 (0.06,2.56) | 0.82 (-0.62,2.25) | 0.05 (-1.32,1.42) | -0.52 (-2.04,1.00) | -0.27 (-1.98,1.44) | -0.87 (-2.67,0.92) | 0.85 (-1.61,3.31) | I |  |  |  |  |  |  |
| 0.29 (-0.96,1.53) | 0.23 (-1.34,1.79) | -0.27 (-1.99,1.45) | -1.04 (-2.63,0.56) | -1.60 (-3.34,0.13) | -1.36 (-3.11,0.40) | -1.95 (-4.00,0.09) | -0.23 (-2.87,2.40) | -1.08 (-2.80,0.63) | J |  |  |  |  |  |
| 0.34 (-1.42,2.11) | 0.28 (-1.32,1.88) | -0.21 (-1.99,1.56) | -0.98 (-2.74,0.77) | -1.55 (-3.45,0.35) | -1.30 (-3.46,0.86) | -1.90 (-4.06,0.26) | -0.18 (-2.84,2.48) | -1.03 (-2.98,0.92) | 0.05 (-2.11,2.21) | K |  |  |  |  |
| 2.55 (0.80,4.31) | 2.49 (0.76,4.22) | 2.00 (0.21,3.79) | 1.23 (-0.37,2.83) | 0.66 (-1.07,2.38) | 0.91 (-1.24,3.06) | 0.31 (-1.04,1.65) | 2.03 (-0.70,4.76) | 1.18 (-0.76,3.13) | 2.26 (0.11,4.42) | 2.21 (-0.06,4.48) | L |  |  |  |
| 0.48 (-1.33,2.29) | 0.42 (-1.31,2.15) | -0.07 (-1.67,1.52) | -0.84 (-2.62,0.93) | -1.41 (-3.32,0.50) | -1.16 (-3.36,1.03) | -1.76 (-3.92,0.39) | -0.04 (-2.78,2.70) | -0.89 (-2.88,1.10) | 0.19 (-2.01,2.39) | 0.14 (-2.05,2.33) | -2.07 (-4.34,0.20) | M |  |  |
| -0.71 (-1.85,0.44) | -0.77 (-1.82,0.29) | -1.26 (-2.37,-0.15) | -2.03 (-3.15,-0.91) | -2.60 (-3.95,-1.24) | -2.35 (-4.04,-0.66) | -2.95 (-4.62,-1.28) | -1.23 (-3.59,1.14) | -2.08 (-3.51,-0.65) | -0.99 (-2.68,0.70) | -1.05 (-2.65,0.56) | -3.26 (-5.07,-1.45) | -1.19 (-2.78,0.41) | N |  |
| -0.48 (-1.88,0.93) | -0.54 (-1.90,0.82) | -1.03 (-2.37,0.31) | -1.80 (-3.21,-0.40) | -2.37 (-3.89,-0.85) | -2.12 (-3.99,-0.25) | -2.72 (-4.31,-1.13) | -1.00 (-3.51,1.52) | -1.85 (-3.30,-0.40) | -0.76 (-2.64,1.11) | -0.82 (-2.78,1.14) | -3.03 (-4.86,-1.20) | -0.96 (-2.89,0.97) | 0.23 (-1.11,1.57) | P |

Appendix 11.3: League table of Summary Estimates for Effective Healing Rate of Acupuncture and Related Therapies on Frozen Shoulder from Network Meta-analysis of 65 Trials

| A |  |  |  |  |  |  |  |  |  |  |  |  |  |
| --- | --- | --- | --- | --- | --- | --- | --- | --- | --- | --- | --- | --- | --- |
| 0.73 (0.55,0.98) | B |  |  |  |  |  |  |  |  |  |  |  |  |
| 0.70 (0.52,0.94) | 0.95 (0.70,1.30) | C |  |  |  |  |  |  |  |  |  |  |  |
| 0.65 (0.51,0.82) | 0.88 (0.66,1.17) | 0.93 (0.68,1.26) | D |  |  |  |  |  |  |  |  |  |  |
| 0.66 (0.44,0.99) | 0.90 (0.56,1.47) | 0.95 (0.58,1.55) | 1.03 (0.66,1.60) | E |  |  |  |  |  |  |  |  |  |
| 0.68 (0.52,0.90) | 0.93 (0.62,1.39) | 0.98 (0.65,1.47) | 1.06 (0.73,1.53) | 1.03 (0.63,1.69) | F |  |  |  |  |  |  |  |  |
| 0.83 (0.52,1.33) | 1.13 (0.67,1.90) | 1.19 (0.70,2.00) | 1.28 (0.80,2.06) | 1.25 (0.68,2.31) | 1.21 (0.70,2.10) | G |  |  |  |  |  |  |  |
| 0.65 (0.45,0.94) | 0.89 (0.59,1.33) | 0.93 (0.60,1.44) | 1.01 (0.67,1.51) | 0.98 (0.57,1.68) | 0.95 (0.60,1.51) | 0.78 (0.44,1.38) | I |  |  |  |  |  |  |
| 0.71 (0.45,1.14) | 0.97 (0.63,1.51) | 1.02 (0.62,1.69) | 1.10 (0.69,1.78) | 1.08 (0.59,1.98) | 1.04 (0.61,1.80) | 0.86 (0.45,1.63) | 1.10 (0.62,1.92) | K |  |  |  |  |  |
| 0.59 (0.40,0.88) | 0.81 (0.51,1.28) | 0.85 (0.53,1.35) | 0.92 (0.62,1.37) | 0.90 (0.51,1.56) | 0.87 (0.53,1.41) | 0.72 (0.50,1.03) | 0.91 (0.54,1.53) | 0.83 (0.46,1.50) | L |  |  |  |  |
| 1.30 (0.96,1.76) | 1.78 (1.29,2.44) | 1.86 (1.27,2.73) | 2.01 (1.45,2.80) | 1.96 (1.19,3.23) | 1.90 (1.26,2.88) | 1.57 (0.91,2.69) | 2.00 (1.27,3.13) | 1.82 (1.21,2.75) | 2.19 (1.36,3.54) | N |  |  |  |
| 0.90 (0.56,1.46) | 1.24 (0.72,2.12) | 1.30 (0.75,2.24) | 1.40 (0.86,2.30) | 1.37 (0.95,1.97) | 1.33 (0.76,2.31) | 1.09 (0.57,2.10) | 1.39 (0.77,2.52) | 1.27 (0.66,2.44) | 1.53 (0.83,2.80) | 0.70 (0.40,1.21) | O |  |  |
| 1.08 (0.79,1.49) | 1.48 (1.02,2.15) | 1.56 (1.08,2.24) | 1.68 (1.24,2.28) | 1.64 (1.00,2.70) | 1.59 (1.04,2.42) | 1.31 (0.82,2.09) | 1.67 (1.09,2.55) | 1.52 (0.89,2.59) | 1.83 (1.19,2.83) | 0.84 (0.56,1.25) | 1.20 (0.69,2.08) | P |  |
| 1.16 (0.74,1.81) | 1.59 (0.96,2.63) | 1.66 (1.00,2.78) | 1.80 (1.14,2.83) | 1.75 (0.97,3.17) | 1.70 (1.01,2.87) | 1.40 (0.75,2.62) | 1.79 (1.02,3.13) | 1.63 (0.87,3.05) | 1.96 (1.10,3.48) | 0.89 (0.53,1.50) | 1.28 (0.68,2.43) | 1.07 (0.64,1.80) | Q |

Appendix 11.4: League table of Summary Estimates for Adverse reactions and shedding of Acupuncture and Related Therapies on Frozen Shoulder from Network Meta-analysis of 65 Trials.

| A |  |  |  |  |  |  |  |
| --- | --- | --- | --- | --- | --- | --- | --- |
| 0.64 (0.02,22.68) | B |  |  |  |  |  |  |
| 1.35 (0.02,88.42) | 2.12 (0.24,18.68) | C |  |  |  |  |  |
| 0.32 (0.00,27.41) | 0.50 (0.03,7.15) | 0.24 (0.01,7.36) | D |  |  |  |  |
| 2.74 (0.24,30.65) | 4.28 (0.06,318.05) | 2.02 (0.02,252.63) | 8.56 (0.05,1353.64) | E |  |  |  |
| 1.23 (0.03,58.81) | 1.92 (0.43,8.58) | 0.91 (0.06,12.76) | 3.83 (0.18,81.27) | 0.45 (0.00,42.88) | F |  |  |
| 0.18 (0.01,4.75) | 0.28 (0.07,1.21) | 0.13 (0.01,1.84) | 0.57 (0.03,11.76) | 0.07 (0.00,3.85) | 0.15 (0.02,1.19) | G |  |
| 0.31 (0.00,40.47) | 0.49 (0.02,13.33) | 0.23 (0.02,2.78) | 0.98 (0.01,68.08) | 0.11 (0.00,26.04) | 0.25 (0.01,9.61) | 1.71 (0.05,63.36) | H |

Appendix 11.5: League table of Summary Estimates for CMS scoring of scapulohumeral periarthritis of shoulder from Network Meta-analysis of 65 Trials..

| A |  |  |  |  |  |  |
| --- | --- | --- | --- | --- | --- | --- |
| -0.83 (-1.91,0.24) | B |  |  |  |  |  |
| 0.62 (-0.40,1.64) | 1.45 (0.11,2.80) | C |  |  |  |  |
| -2.58 (-3.95,-1.21) | -1.75 (-2.85,-0.65) | -3.20 (-4.65,-1.76) | I |  |  |  |
| -2.11 (-3.98,-0.23) | -1.28 (-3.24,0.69) | -2.73 (-4.41,-1.05) | 0.48 (-1.45,2.40) | K |  |  |
| 2.28 (0.96,3.61) | 3.12 (1.66,4.57) | 1.66 (0.63,2.70) | 4.87 (3.47,6.26) | 4.39 (3.06,5.72) | N |  |
| 0.54 (-0.91,2.00) | 1.37 (-0.06,2.80) | -0.08 (-1.41,1.25) | 3.12 (1.91,4.33) | 2.65 (0.97,4.33) | -1.74 (-2.77,-0.72) | P |

Appendix 12 Adverse reactions and shedding

| Study | Adverse reactions and shedding | | |
| --- | --- | --- | --- |
|  | Intervention | Control | |
| Li He 2016 | One patient experienced needle syncope, and another patient had abnormal liver function. | | One patient dropped out midway |
| Pei Zheng Lei 2013 | One patient withdrew from the study during the first course of treatment due to the occurrence of general swelling | | No dropouts occurred. |
| Qiong Zhang 2018 | Among them, there were 2 dropouts in Control Group 1. One patient developed local skin depigmentation after the 2nd treatment, and the other developed local muscle induration accompanied by urticaria after the 3rd treatment. Both cases were classified as dropouts, with their efficacy at that time evaluated and included in the statistics. | | No dropouts occurred. |
| TianYi Zhou 2023 | No dropouts occurred. | | One patient in the Acupuncture Group dropped out due to personal reasons. |

Appendix 13 Subgroup Analysis

Appendix 13.1 SUCRA of n≤14days

| Treatment | SUCRA | PrBest | MeanRank |
| --- | --- | --- | --- |
| A | 35.6 | 0 | 10.7 |
| B | 63.7 | 0.3 | 6.4 |
| C | 28 | 0 | 11.8 |
| D | 76.9 | 1.8 | 4.5 |
| E | 63.9 | 0.3 | 6.4 |
| F | 41.3 | 0 | 9.8 |
| G | 59.6 | 1.9 | 7.1 |
| I | 83.3 | 26.2 | 3.5 |
| J | 79 | 21.5 | 4.2 |
| K | 61 | 2.1 | 6.9 |
| L | 90.1 | 44.5 | 2.5 |
| M | 44.6 | 1.3 | 9.3 |
| N | 12.7 | 0 | 14.1 |
| O | 31.7 | 0.1 | 11.2 |
| P | 18.5 | 0 | 13.2 |
| Q | 10.1 | 0 | 14.5 |

Appendix 13.2 League Table of n<14days

| A |  |  |  |  |  |  |  |  |  |  |  |  |  |  |  |
| --- | --- | --- | --- | --- | --- | --- | --- | --- | --- | --- | --- | --- | --- | --- | --- |
| 0.89 (0.76,1.03) | B |  |  |  |  |  |  |  |  |  |  |  |  |  |  |
| 1.04 (0.85,1.28) | 1.18 (0.95,1.46) | C |  |  |  |  |  |  |  |  |  |  |  |  |  |
| 0.82 (0.70,0.97) | 0.93 (0.74,1.16) | 0.79 (0.61,1.02) | D |  |  |  |  |  |  |  |  |  |  |  |  |
| 0.89 (0.78,1.02) | 1.00 (0.83,1.21) | 0.85 (0.70,1.04) | 1.08 (0.89,1.32) | E |  |  |  |  |  |  |  |  |  |  |  |
| 0.98 (0.84,1.14) | 1.10 (0.89,1.37) | 0.94 (0.73,1.20) | 1.19 (0.95,1.49) | 1.10 (0.90,1.35) | F |  |  |  |  |  |  |  |  |  |  |
| 0.90 (0.72,1.13) | 1.02 (0.78,1.33) | 0.86 (0.64,1.16) | 1.10 (0.83,1.45) | 1.01 (0.78,1.32) | 0.92 (0.70,1.21) | G |  |  |  |  |  |  |  |  |  |
| 0.76 (0.56,1.03) | 0.86 (0.62,1.18) | 0.73 (0.52,1.03) | 0.93 (0.66,1.30) | 0.86 (0.62,1.18) | 0.78 (0.56,1.09) | 0.84 (0.58,1.23) | I |  |  |  |  |  |  |  |  |
| 0.79 (0.58,1.07) | 0.89 (0.63,1.25) | 0.75 (0.52,1.09) | 0.95 (0.67,1.35) | 0.88 (0.63,1.24) | 0.80 (0.57,1.13) | 0.87 (0.60,1.27) | 1.03 (0.67,1.59) | J |  |  |  |  |  |  |  |
| 0.89 (0.70,1.13) | 1.01 (0.82,1.23) | 0.85 (0.65,1.12) | 1.08 (0.81,1.44) | 1.00 (0.77,1.30) | 0.91 (0.69,1.21) | 0.99 (0.71,1.37) | 1.17 (0.81,1.69) | 1.13 (0.77,1.67) | K |  |  |  |  |  |  |
| 0.72 (0.53,0.97) | 0.81 (0.58,1.13) | 0.69 (0.48,0.98) | 0.87 (0.68,1.12) | 0.81 (0.59,1.11) | 0.73 (0.52,1.03) | 0.80 (0.55,1.16) | 0.94 (0.62,1.44) | 0.91 (0.59,1.41) | 0.81 (0.55,1.18) | L |  |  |  |  |  |
| 0.97 (0.72,1.30) | 1.09 (0.81,1.48) | 0.93 (0.75,1.15) | 1.18 (0.84,1.64) | 1.09 (0.81,1.46) | 0.99 (0.71,1.38) | 1.07 (0.74,1.55) | 1.27 (0.85,1.90) | 1.23 (0.81,1.88) | 1.09 (0.77,1.54) | 1.35 (0.89,2.04) | M |  |  |  |  |
| 1.13 (0.97,1.32) | 1.28 (1.10,1.49) | 1.08 (0.90,1.30) | 1.37 (1.10,1.72) | 1.27 (1.06,1.53) | 1.16 (0.93,1.44) | 1.25 (0.96,1.65) | 1.48 (1.09,2.02) | 1.44 (1.02,2.03) | 1.27 (1.02,1.58) | 1.58 (1.13,2.21) | 1.17 (0.88,1.54) | N |  |  |  |
| 1.03 (0.83,1.28) | 1.16 (0.89,1.50) | 0.98 (0.74,1.30) | 1.25 (1.01,1.54) | 1.16 (0.94,1.43) | 1.05 (0.80,1.37) | 1.14 (0.83,1.56) | 1.35 (0.93,1.95) | 1.31 (0.90,1.91) | 1.15 (0.84,1.58) | 1.43 (1.03,1.99) | 1.06 (0.75,1.51) | 0.91 (0.70,1.18) | O |  |  |
| 1.10 (0.91,1.33) | 1.24 (1.01,1.53) | 1.05 (0.83,1.34) | 1.34 (1.04,1.72) | 1.24 (0.99,1.55) | 1.13 (0.88,1.44) | 1.22 (0.91,1.64) | 1.45 (1.10,1.90) | 1.40 (0.98,2.01) | 1.24 (0.94,1.62) | 1.53 (1.08,2.19) | 1.14 (0.83,1.57) | 0.97 (0.82,1.16) | 1.07 (0.81,1.43) | P |  |
| 1.22 (0.91,1.62) | 1.38 (0.99,1.90) | 1.17 (0.82,1.66) | 1.48 (1.06,2.06) | 1.37 (1.00,1.88) | 1.25 (0.90,1.73) | 1.35 (0.94,1.94) | 1.60 (1.05,2.43) | 1.55 (1.02,2.36) | 1.37 (0.94,1.98) | 1.70 (1.12,2.58) | 1.26 (0.83,1.90) | 1.08 (0.78,1.49) | 1.19 (0.83,1.70) | 1.11 (0.78,1.56) | Q |

Appendix 13.3 SUCRA of 14<n≤28days

| Treatm~t | SUCRA | PrBest | MeanRank |
| --- | --- | --- | --- |
| A | 22.2 | 0 | 11.9 |
| B | 31.9 | 0 | 10.5 |
| C | 34.9 | 0 | 10.1 |
| D | 59.7 | 0 | 6.6 |
| E | 95.6 | 59.4 | 1.6 |
| F | 48.1 | 0.1 | 8.3 |
| G | 45.7 | 0.7 | 8.6 |
| I | 68 | 4.1 | 5.5 |
| K | 58.2 | 12.5 | 6.9 |
| L | 75.9 | 6.4 | 4.4 |
| M | 59.7 | 8.2 | 6.6 |
| N | 4 | 0 | 14.4 |
| O | 62 | 4.3 | 6.3 |
| P | 27.7 | 0 | 11.1 |
| Q | 56.3 | 4.2 | 7.1 |

Appendix 13.4 League Table of 14<n≤28days

| A |  |  |  |  |  |  |  |  |  |  |  |  |  |  |
| --- | --- | --- | --- | --- | --- | --- | --- | --- | --- | --- | --- | --- | --- | --- |
| 0.98 (0.87,1.10) | B |  |  |  |  |  |  |  |  |  |  |  |  |  |
| 0.97 (0.87,1.07) | 0.99 (0.87,1.13) | C |  |  |  |  |  |  |  |  |  |  |  |  |
| 0.91 (0.82,1.00) | 0.93 (0.83,1.03) | 0.94 (0.84,1.05) | D |  |  |  |  |  |  |  |  |  |  |  |
| 0.76 (0.67,0.86) | 0.78 (0.68,0.90) | 0.79 (0.69,0.90) | 0.84 (0.74,0.96) | E |  |  |  |  |  |  |  |  |  |  |
| 0.93 (0.85,1.03) | 0.96 (0.82,1.12) | 0.97 (0.84,1.12) | 1.03 (0.90,1.18) | 1.23 (1.05,1.44) | F |  |  |  |  |  |  |  |  |  |
| 0.94 (0.79,1.12) | 0.96 (0.79,1.17) | 0.97 (0.80,1.17) | 1.04 (0.87,1.24) | 1.23 (1.00,1.51) | 1.00 (0.82,1.23) | G |  |  |  |  |  |  |  |  |
| 0.87 (0.74,1.03) | 0.90 (0.77,1.05) | 0.90 (0.76,1.08) | 0.96 (0.82,1.14) | 1.15 (0.95,1.39) | 0.94 (0.77,1.13) | 0.93 (0.75,1.16) | I |  |  |  |  |  |  |  |
| 0.89 (0.66,1.20) | 0.92 (0.68,1.23) | 0.93 (0.68,1.26) | 0.99 (0.73,1.33) | 1.17 (0.86,1.60) | 0.96 (0.70,1.31) | 0.95 (0.68,1.34) | 1.02 (0.74,1.42) | K |  |  |  |  |  |  |
| 0.85 (0.74,0.99) | 0.87 (0.74,1.04) | 0.88 (0.75,1.04) | 0.94 (0.81,1.09) | 1.12 (0.93,1.34) | 0.91 (0.77,1.09) | 0.91 (0.79,1.04) | 0.98 (0.80,1.20) | 0.95 (0.69,1.32) | L |  |  |  |  |  |
| 0.89 (0.70,1.14) | 0.91 (0.71,1.18) | 0.92 (0.74,1.15) | 0.98 (0.77,1.26) | 1.17 (0.91,1.51) | 0.95 (0.74,1.24) | 0.95 (0.71,1.27) | 1.02 (0.77,1.35) | 1.00 (0.69,1.45) | 1.05 (0.79,1.38) | M |  |  |  |  |
| 1.10 (0.96,1.24) | 1.12 (1.00,1.27) | 1.13 (0.98,1.31) | 1.21 (1.07,1.37) | 1.44 (1.23,1.69) | 1.17 (1.00,1.38) | 1.17 (0.95,1.43) | 1.25 (1.04,1.51) | 1.23 (0.94,1.60) | 1.28 (1.07,1.54) | 1.23 (0.95,1.60) | N |  |  |  |
| 0.89 (0.73,1.08) | 0.91 (0.73,1.13) | 0.92 (0.74,1.13) | 0.98 (0.80,1.21) | 1.17 (0.96,1.41) | 0.95 (0.77,1.18) | 0.95 (0.73,1.23) | 1.02 (0.79,1.30) | 0.99 (0.70,1.41) | 1.04 (0.82,1.32) | 1.00 (0.74,1.35) | 0.81 (0.65,1.01) | O |  |  |
| 0.99 (0.86,1.15) | 1.02 (0.87,1.19) | 1.03 (0.88,1.19) | 1.09 (0.95,1.25) | 1.30 (1.09,1.54) | 1.06 (0.89,1.27) | 1.06 (0.88,1.26) | 1.13 (0.95,1.36) | 1.11 (0.81,1.52) | 1.16 (0.98,1.38) | 1.11 (0.85,1.44) | 0.90 (0.76,1.07) | 1.12 (0.88,1.41) | P |  |
| 0.91 (0.73,1.12) | 0.93 (0.75,1.16) | 0.94 (0.75,1.17) | 1.00 (0.83,1.21) | 1.19 (0.95,1.49) | 0.97 (0.77,1.23) | 0.97 (0.74,1.25) | 1.04 (0.81,1.33) | 1.01 (0.71,1.44) | 1.06 (0.83,1.35) | 1.02 (0.75,1.38) | 0.83 (0.66,1.04) | 1.02 (0.77,1.35) | 0.91 (0.72,1.15) | Q |

Appendix13.5 SUCRA of n>28days

| Treatment | SUCRA | PrBest | MeanRank |
| --- | --- | --- | --- |
| A | 66.8 | 1.4 | 3.3 |
| B | 34.5 | 0 | 5.6 |
| C | 60.7 | 0 | 3.8 |
| D | 35.1 | 0 | 5.5 |
| E | 57.9 | 49.6 | 3.9 |
| M | 85.5 | 48.9 | 2 |
| O | 42.7 | 0 | 5 |
| P | 16.7 | 0 | 6.8 |

Appendix13.6 League Table of n>28days

| A |  |  |  |  |  |  |  |
| --- | --- | --- | --- | --- | --- | --- | --- |
| 1.33 (1.02,1.73) | B |  |  |  |  |  |  |
| 1.06 (0.86,1.30) | 0.80 (0.67,0.95) | C |  |  |  |  |  |
| 1.35 (0.98,1.86) | 1.02 (0.76,1.37) | 1.27 (1.00,1.63) | D |  |  |  |  |
| 0.87 (0.00,2.58e+11) | 0.65 (0.00,1.94e+11) | 0.82 (0.00,2.43e+11) | 0.64 (0.00,1.91e+11) | E |  |  |  |
| 0.77 (0.60,1.00) | 0.58 (0.46,0.74) | 0.73 (0.62,0.86) | 0.57 (0.43,0.77) | 0.89 (0.00,2.66e+11) | M |  |  |
| 1.20 (0.00,3.57e+11) | 0.90 (0.00,2.69e+11) | 1.13 (0.00,3.36e+11) | 0.89 (0.00,2.64e+11) | 1.38 (1.16,1.65) | 1.55 (0.00,4.61e+11) | O |  |
| 1.53 (1.15,2.04) | 1.15 (0.89,1.50) | 1.44 (1.18,1.76) | 1.13 (0.98,1.30) | 1.77 (0.00,5.26e+11) | 1.98 (1.53,2.56) | 1.28 (0.00,3.80e+11) | P |
